# Up-regulated expression of two-pore domain K^+^ channels, KCNK1 and KCNK2, is involved in the proliferation and migration of pulmonary arterial smooth muscle cells in pulmonary arterial hypertension

**DOI:** 10.3389/fcvm.2024.1343804

**Published:** 2024-02-12

**Authors:** Natsumi Shima, Aya Yamamura, Moe Fujiwara, Taiki Amano, Kazuyuki Matsumoto, Taiga Sekine, Haruka Okano, Rubii Kondo, Yoshiaki Suzuki, Hisao Yamamura

**Affiliations:** ^1^ Department of Molecular and Cellular Pharmacology, Graduate School of Pharmaceutical Sciences, Nagoya City University, Nagoya, Japan; ^2^ Department of Physiology, Aichi Medical University, Nagakute, Japan

**Keywords:** pulmonary hypertension, KCNK1, KCNK2, two-pore domain potassium channel, vascular remodeling, proliferation, migration, JNK

## Abstract

**Background:**

Pulmonary arterial hypertension (PAH) is a severe and rare disease in the cardiopulmonary system. Its pathogenesis involves vascular remodeling of the pulmonary artery, which results in progressive increases in pulmonary arterial pressure. Chronically increased pulmonary arterial pressure causes right ventricular hypertrophy and subsequent right heart failure. Pulmonary vascular remodeling is attributed to the excessive proliferation and migration of pulmonary arterial smooth muscle cells (PASMCs), which are induced by enhanced Ca^2+^ signaling following the up-/down-regulation of ion channel expression.

**Objectives:**

In the present study, the functional expression of two-pore domain potassium KCNK channels was investigated in PASMCs from idiopathic PAH (IPAH) patients and experimental pulmonary hypertensive (PH) animals.

**Results:**

In IPAH-PASMCs, the expression of KCNK1/TWIK1 and KCNK2/TREK1 channels was up-regulated, whereas that of KCNK3/TASK1 and KCNK6/TWIK2 channels was down-regulated. The similar up-regulated expression of KCNK1 and KCNK2 channels was observed in the pulmonary arterial smooth muscles of monocrotaline-induced PH rats, Sugen 5416/hypoxia-induced PH rats, and hypoxia-induced PH mice. The facilitated proliferation of IPAH-PASMCs was suppressed by the KCNK channel blockers, quinine and tetrapentylammonium. The migration of IPAH-PASMCs was also suppressed by these channel blockers. Furthermore, increases in the proliferation and migration were inhibited by the siRNA knockdown of KCNK1 or KCNK2 channels. The siRNA knockdown also caused membrane depolarization and subsequent decrease in cytosolic [Ca^2+^]. The phosphorylated level of c-Jun N-terminal kinase (JNK) was elevated in IPAH-PASMCs compared to normal-PASMCs. The increased phosphorylation was significantly reduced by the siRNA knockdown of KCNK1 or KCNK2 channels.

**Conclusion:**

Collectively, these findings indicate that the up-regulated expression of KCNK1 and KCNK2 channels facilitates the proliferation and migration of PASMCs via enhanced Ca^2+^ signaling and JNK signaling pathway, which is associated with vascular remodeling in PAH.

## 1 Introduction

Pulmonary arterial hypertension (PAH), which is known as clinical classification Group 1 of pulmonary hypertension (PH), is a rare and life-threatening disease in the cardiovascular/respiratory systems. It is characterized by the vascular remodeling of pulmonary arterioles (<500 μm in diameter). These pathological events induce constitutive increases in pulmonary arterial pressure. Chronically increased pulmonary arterial pressure causes right ventricular hypertrophy, and ultimately, right heart failure with high mortality (1). PAH has been categorized by its underlying etiologies: idiopathic (IPAH, 46.2%), associated (45.4%), drug/toxin-induced (5.3%), and heritable (2.7%) PAH (2). The etiological causes of IPAH remain unknown or there is no family history. Heritable PAH causes familial mutations in the genes encoding activin A receptor-like type 1, bone morphogenetic protein receptor type 2, caveolin 1, endoglin, mothers against decapentaplegic homolog 9, and two-pore domain potassium channel subfamily K member 3 (KCNK3/TASK1) (3). Associated PAH occurs with congenital heart disease, connective tissue disease, human immunodeficiency virus infection, schistosomiasis, and portal hypertension (1).

In PAH, the progression of irreversible vascular remodeling are predominantly caused by the facilitated proliferation and migration of pulmonary arterial smooth muscle cells (PASMCs) composing the medial layer of the pulmonary artery. The proliferation and migration of PASMCs are elicited by a rise in cytosolic [Ca^2+^] ([Ca^2+^]_cyt_), which is regulated by Ca^2+^ influx through Ca^2+^-permeable ion channels: e.g., voltage-dependent Ca^2+^ channels (VDCCs), receptor-operated Ca^2+^ (ROC) channels, and store-operated Ca^2+^ (SOC) channels. It is also caused by Ca^2+^ release from intracellular Ca^2+^ stores: e.g., the sarcoplasmic reticulum (4, 5). The activity of K^+^ and Cl^−^ channels has also been shown to participate in the modulation of [Ca^2+^]_cyt_ through the membrane potential in PASMCs (6, 7).

The two-pore domain potassium KCNK channel family contains 15 genes (KCNK1 to 18, except for 8, 11, and 14) that are classified into six subfamilies based on sequence similarity and functional resemblance: TWIK, TREK, TASK, TALK, THIK, and TRESK (8–10). The KCNK subunit has four transmembrane structures containing two pore-forming regions that form a functional ion channel as a homomeric or heteromeric dimer. KCNK channels produce background or leak K^+^ currents, thereby maintaining the resting membrane potential and [Ca^2+^]_cyt_ in several types of cells. Pharmacologically, KCNK channels are sensitive to lipids, temperature, membrane stretch, pH, and volatile anesthetics. In the pathological profile, KCNK channels are associated with depression, epilepsy, cardiac arrhythmia, nociception, and cancers (8, 9). Furthermore, missense mutations in the KCNK3/TASK1 gene are associated with heritable PAH (3). Limited information is, however, available on the involvement of other KCNK channels in PAH.

Mitogen-activated protein kinases (MAPKs) are a group of serine/threonine protein kinases that play a pivotal role in regulating the growth, proliferation, differentiation, migration, and apoptosis of vascular myocytes (4, 5). MAPKs include extracellular signal-regulated protein kinase 1/2, p38 MAPK, and c-Jun N-terminal kinase (JNK), which are activated by mitogen, hormones, growth factors, cytokines, and environmental stresses (11). Previous studies reported the activation of JNK in the pulmonary artery of experimental PH animals (12, 13) and PAH patients (14) and also in hypoxia-treated PASMCs (15). Among the three isoforms of JNK (JNK1 to 3), JNK2 is predominantly responsible for vascular remodeling in hypoxia-induced PH (16, 17). Therefore, JNK signaling has been supposed to participate in the process of vascular remodeling in PAH.

In the present investigation, the expression of KCNK channels in PASMCs from IPAH patients and experimental PH animals (monocrotaline (MCT)-induced PH rats, Sugen 5416/hypoxia (SuHx)-induced PH rats, and hypoxia-induced PH mice) was analyzed using quantitative real-time PCR (qPCR), Western blotting, and immunohistochemical staining. The contribution of KCNK channels to the enhanced proliferation of IPAH-PASMCs was assessed by WST-8 and bromodeoxyuridine (BrdU) incorporation assays. The role of KCNK channels in the migration of IPAH-PASMCs was investigated by Transwell assays, and their involvement in the phosphorylation of JNK in IPAH-PASMCs was also evaluated. In addition, the involvement of KCNK channels in the regulation of the resting membrane potential and [Ca^2+^]_cyt_ in IPAH-PASMCs was examined by fluorescence DiBAC_4_(3) and fura-2 imaging, respectively. The present investigation clearly showed that the expression of KCNK1/TWIK1 and KCNK2/TREK1 channels was up-regulated in PASMCs from IPAH patients, MCT-PH rats, SuHx-PH rats, and hypoxia-PH mice. The proliferation and migration of IPAH-PASMCs were inhibited by KCNK channel blockers and by the siRNA knockdown of KCNK1 or KCNK2 channels. These siRNA knockdown also caused membrane depolarization and decreased resting [Ca^2+^]_cyt_. The phosphorylation level of JNK was reduced by the siRNA knockdown of KCNK1 or KCNK2 channels. Collectively, these findings indicate that the up-regulated expression of KCNK1 and KCNK2 channels is associated with vascular remodeling through enhanced Ca^2+^ signaling and JNK signaling pathway in PAH.

## 2 Materials and methods

### 2.1 Cell culture

PASMCs (passages 5–10) from normal subjects (Lonza, Basel, Switzerland) and IPAH patients (kindly offered by Prof. Jason X.-J. Yuan) (18, 19) were cultivated in Medium 199 supplemented with fetal bovine serum (FBS, 10%; Thermo Fisher Scientific, Waltham, MA, USA), D-valine (50 μg/ml; MilliporeSigma, Burlington, MA, USA), endothelial cell growth supplement (20 μg/ml; BD Biosciences, Franklin Lakes, NJ, USA), penicillin G (100 U/ml), and streptomycin (100 μg/ml; Fujifilm Wako Pure Chemical, Osaka, Japan) at 37°C.

### 2.2 Experimental PH animals

Animal experiments were approved by the Ethics Committees of Nagoya City University (H30-P-1) and Aichi Medical University (2019–15). To generate MCT-PH rats (20–22), rats (Sprague-Dawley, male, 4 weeks old, 100–110 g; Japan SLC, Hamamatsu, Japan) were subcutaneously injected with vehicle (saline) or MCT (60 mg/kg; MilliporeSigma) and bred for 3 weeks (23). To obtain SuHx-PH rats (20–22), rats (Sprague-Dawley, male, 6 weeks old, 220–250 g; Japan SLC) were subcutaneously injected with vehicle (saline) or Sugen 5416 (20 mg/kg; MedChemExpress, Monmouth Junction, NJ, USA) and bred in a hypoxic chamber (10% O_2_) with a ProOx110 O_2_ controller (Biospherix, Parish, NY, USA) for 3 weeks and thereafter in a normoxic chamber for 2 weeks. Regarding hypoxia-PH mice (20–22), mice (C57BL/6, male, 8 weeks old, 18–23 g; Japan SLC) were bred in a normoxic or hypoxic (10% O_2_) chamber for 4 weeks. Rats/mice were anesthetized by an intraperitoneal injection of ketamine (100 mg/kg) and xylazine (26 mg/kg). The pulmonary artery (the first to third branches) and right ventricle were dissected in Ca^2+^/Mg^2+^-free Krebs solution (in mM; 112 NaCl, 4.7 KCl, 25 NaHCO_3_, 1.2 KH_2_PO_4_, 14 glucose, and pH 7.4 by gassing with 95% O_2_/5% CO_2_). The endothelium layer of the pulmonary artery was stripped out by water flow.

### 2.3 qPCR

Total RNA was extracted using RNAiso Plus (Takara Bio, Kusatsu, Japan) and reverse transcribed to cDNA using the ReverTra Ace qPCR RT Master Mix (Toyobo, Osaka, Japan) (24). A qPCR analysis was carried out using SYBR *Premix Ex Taq* (Takara Bio) by the LightCycler 96 qPCR system (Roche Diagnostics, Basel, Switzerland). Specific primers were designed as shown in Table 1.

**Table 1:** Specific primers of KCNK channel genes for qPCR.

Gene	GenBank accession number	Sense primer	Antisense primer
Human			
KCNK1 (TWIK1)	NM_002245	GAACTGGGACTTCACCTCCG	AGATGATGCAGAAGGCCTTACC
KCNK2 (TREK1)	NM_014217	AACATCTCACCACGCACAGAAG	TCCACTTTGGCAATTCCTTTTC
KCNK3 (TASK1)	NM_002246	ATCACCGTCATCACCACCATC	AACATGCAGAACACCTTGCC
KCNK4 (TRAAK)	NM_033310	TGGCATCGGTCACATTGAAG	GTGGGCGTGAGGACAAAGAG
KCNK5 (TASK2)	NM_003740	CCATCACAGGGAACCAGACC	CCCCGAAGAGACCATAGAAAAC
KCNK6 (TWIK2)	NM_004823	CCCTCTACAAGGTGCTGGTC	CATTGAAACTGGCAGGGCAC
KCNK7	NM_033347	TTCCCTCAGCCCTGCTCTTC	TGGCCACGAGAGCTAAGGAG
KCNK9 (TASK3)	NM_001282534	TGCTGAAGAGAGGGCATCC	AGGTGCAGGAGCACACAGAC
KCNK10 (TREK2)	NM_021161	TTGTTGGCCTTGCCTACTTTG	GAACTCAGCCGTGACATTGG
KCNK12 (THIK2)	NM_022055	TCGTCACCTTCAGCACCATC	GAGCGAGTAAATGCAGCACAC
KCNK13 (THIK1)	NM_022054	ACTTCACCGGCGCCTTCTAC	GTGCTGGAACACCCAACAAG
KCNK15 (TASK5)	NM_022358	CCTTCCTCAACCTGGTGGTC	GCACGTGGCAGAAGACAGAG
KCNK16 (TALK1)	NM_032115	AGGGACCAGTTTCAGTTGGAG	TGCTGGGGTTGGTAGAGTTG
KCNK17 (TALK2)	NM_031460	CGACAAGTGGGAGCTGTTG	CTGGTGGTGTTGCTGAGGAG
KCNK18 (TRESK)	NM_181840	TTTTCTGCTGCACGGTGTTC	ATGTCGCCTGTGTCCGTGAG
ACTB (β-actin)	NM_001101	AGGCCAACCGCGAGAAGATG	GCCAGAGGCGTACAGGGATA
Rat			
Kcnk1 (TWIK1)	NM_021688	TGGAGGCCAGCAATTATGGAG	ACCGTGTGGCCATAGCCTG
Kcnk2 (TREK1)	NM_172041	GCGATTATGTGGCAGGTGGG	CATTGGCTGTCCACTCAGCG
Kcnk3 (TASK1)	NM_033376	CGTCATCACCACAATCGGCTATG	GTTGATGCGTTCACCCAGGC
Kcnk6 (TWIK2)	NM_053806	AGTCACCACCGTGGGCTATG	GTAGCATGGTGATAGGCACGC
Actb (β-actin)	NM_031144	AGGCCAACCGTGAAAAGATG	ACCAGAGGCATACAGGGACA

### 2.4 Western blotting

Protein fraction was extracted using RIPA buffer (for human PASMCs) and T-PER Tissue Protein Extraction Reagent (for rat/mouse pulmonary arterial smooth muscles (PASMs); Thermo Fisher Scientific) (23). Extracted protein (20 μg/lane) was applied to an acrylamide gel (8%) and transferred to an Immobilon-P PVDF membrane (MilliporeSigma). The membrane was blocked with Tris-buffered saline containing bovine serum albumin (5%) and Tween 20 (0.1%; MilliporeSigma) at room temperature (25°C) for 3 h and then treated with a primary antibody for KCNK1 (1:800; APC-110), KCNK2 (1:800; APC-047), KCNK3 (1:800; APC-024), KCNK6 (1:800; APC-040, Alomone Labs, Jerusalem, Israel), JNK (1:1000; #9252), or phospho (*p*)-JNK (1:1000; #4668, Cell Signaling Technology, Danvers, MA, USA) at 4°C for 18 h. Immunoblotted membranes were then exposed to an anti-rabbit HRP-conjugated IgG secondary antibody (1:5000; #170-6515, Bio-Rad Laboratories, Hercules, CA, USA) at room temperature for 1 h. Blotting signals were detected using an ImmunoStar LD reagent (Fujifilm Wako Pure Chemical) and observed with the Imager 600 system (GE HealthCare Technologies, Chicago, IL, USA). Protein expression levels were normalized using anti-β-actin (1:5000; A5316, MilliporeSigma) and anti-mouse HRP-conjugated IgG (1:10000; #170-6516, Bio-Rad Laboratories) antibodies.

### 2.5 Immunohistochemical staining

The lungs of MCT-PH rats were fixed with paraformaldehyde (4%; MilliporeSigma) in PBS. Paraffin-embedded sections from lung lobes were prepared by the Biopathology Institute (Oita, Japan). They were deparaffinized and heat-induced epitope retrieval was carried out with Tris-HCl (10 mM, pH 9.0) containing EDTA (1 mM) at 115°C for 10 min. As the first step, sections were treated with a KCNK1 or KCNK2 antibody (1:100) using an ImmPRESS HRP reagent kit (Vector Laboratories, Burlingame, CA, USA) at room temperature for 1 h. After washing twice in PBS, they were covered with a secondary antibody contained in the ImmPRESS HRP reagent kit at room temperature for 30 min and rinsed twice with PBS. They were then treated with Fluorescein (1:200) in 1×plus amplification diluent (Akoya Biosciences, Marlborough, MA, USA) at room temperature for 10 min. After heating in a microwave for 1 min and washing with PBS, the same sections were treated with an *α*-smooth muscle actin (*α*-SMA) antibody (1:1000; #19245, Cell Signaling Technology), the ImmPRESS HRP reagent kit, and Cyanine 3 (1:400) using the same protocol as the first step. They were also stained with 4’,6-diamidino-2-phenylindole (DAPI; Dojindo Laboratories, Kumamoto, Japan). Immunohistochemical images were obtained using the Aperio CS2 image capture device (Leica Biosystems, Wetzlar, Germany).

### 2.6 Cell proliferation assay

Human PASMCs (3 × 10^3^ cells/well) were seeded on a 96-well plate (Falcon #353075, Corning, Corning, NY, USA) and cultured at 37°C for 6 h (23). Thereafter, they were treated with culture medium containing FBS (10%) and the vehicle [dimethyl sulfoxide (DMSO)] or drug for 48 h. Cell viability was assessed using Cell Counting Kit-8 (Dojindo Laboratories) based on the WST-8 assay. Cell proliferation was assessed using the Cell Proliferation ELISA, BrdU kit (Roche Diagnostics).

### 2.7 Cell migration assay

Human PASMCs (5 × 10^4^ cells/well) were seeded on a 24-well Transwell insert with a membrane pore size of 8 μm (#3422, Corning) (23). Thereafter, they were treated with culture medium containing FBS (1%) in the upper chamber and that containing FBS (10%) and the vehicle (DMSO) or drug in the lower chamber for 24 h. Transwell inserts were fixed in paraformaldehyde (4%) and stained with crystal violet (1%; Fujifilm Wako Pure Chemical). The number of migratory cells was counted from digital images of Transwell inserts using the SMZ1270 stereomicroscope system equipped with a DS-Vi1 color microscope camera and NIS-Elements imaging software (Nikon, Tokyo, Japan).

### 2.8 siRNA knockdown

Human PASMCs (3 × 10^3^ cells/well) were seeded on a 96-well plate (Falcon #353075, Corning) and cultured at 37°C for 6 h. Thereafter, they were transiently transfected with universal negative control, KCNK1 ((+) UUGCCAUGUUGGUAGUUCUdTdT and (-) AGAACUACCAACAUGGCAAdTdT), or KCNK2 ((+) GGAAACACCUCCAAUCAAAdTdT and (-) UUUGAUUGGAGGUGUUUCCdTdT) siRNA construct (20 nM; Nippon Gene, Tokyo, Japan) using Lipofectamine RNAiMax transfection reagent (Thermo Fisher Scientific). The culture medium was replaced with siRNA-free medium 12–24 h after transfection. Experiments using siRNA were performed 48 h after transfection.

### 2.9 Measurement of the membrane potential

Human PASMCs were incubated with the voltage-sensitive fluorescent dye, DiBAC_4_(3) (100 nM; Dojindo Laboratories), at room temperature for 30 min. DiBAC_4_(3) at the same concentration was added to the extracellular solution during measurements. Fluorescent signals were measured using the A1R confocal fluorescence imaging system equipped with an ECLIPSE Ti inverted microscope, a Plan Apo VC objective lens (20×/0.75), NIS-Elements imaging software (Nikon), and a solid-state 488-nm laser (Coherent, Santa Clara, CA, USA). PASMCs were illuminated at a 488-nm wavelength, and the fluorescent emissions (>520 nm) were obtained every 5 s. Membrane potential is presented as F/F_140K_, where F is the fluorescence intensity and F_140K_ is the maximum fluorescence intensity in the 140-mM K^+^ HEPES-buffered solution (theoretically 0 mV). Standard HEPES-buffered solution was used as an extracellular solution (in mM): 137 NaCl, 5.9 KCl, 2.2 CaCl_2_, 1.2 MgCl_2_, 14 glucose, 10 HEPES, and pH 7.4 with NaOH. In the 140-mM K^+^ HEPES-buffered solution, the concentrations of NaCl and KCl in standard HEPES-buffered solution were changed to 2.9 and 140 mM, respectively.

### 2.10 Measurement of [Ca^2+^]_cyt_

Human PASMCs were loaded with fura-2 acetoxymethyl ester (fura-2/AM, 10 μM; Thermo Fisher Scientific) at room temperature for 30 min. [Ca^2+^]_cyt_ measurements were performed using the fluorescence imaging system equipped with an ECLIPSE Ti2 inverted microscope, a S FL objective lens (20×/0.75), NIS-Elements imaging software (Nikon), a pE-340^fura^ LED illuminator (CoolLED, Hampshire, UK), and a C9100-12 EM-CCD digital camera (Hamamatsu Photonics, Hamamatsu, Japan). PASMCs were illuminated at 340-/380-nm wavelengths, and the fluorescent emissions (510/80 nm) were obtained every 5 s. The fura-2 signal is presented as the fluorescence ratio (F_340_/F_380_). Standard HEPES-buffered solution was used as an extracellular solution.

### 2.11 Drugs

Pharmacological reagents were obtained from Fujifilm Wako Pure Chemical, except for EDTA, HEPES (Dojindo Laboratories), and quinine (MilliporeSigma). Quinine and tetrapentylammonium (TPA) were dissolved in DMSO at concentrations of 150 and 100 mM, respectively, as a stock solution.

### 2.12 Statistical analysis

Pooled data are shown as the means ± S.E. The significance of differences between two groups was examined using the non-parametric Mann–Whitney *U* test (*n* < 10) or Student's *t*-test (*n *≥ 10) using BellCurve software (Social Survey Research Information, Tokyo, Japan). The significance of differences among groups was assessed by Scheffé's or Steel's test after non-parametric Kruskal-Wallis test (*n* < 10) or Scheffé's test after an analysis of variance (ANOVA) (*n* ≥ 10) using the same software.

## 3 Results

### 3.1 Up-regulation of KCNK1 and KCNK2 channel expression in IPAH-PASMCs

**Figure 1:**
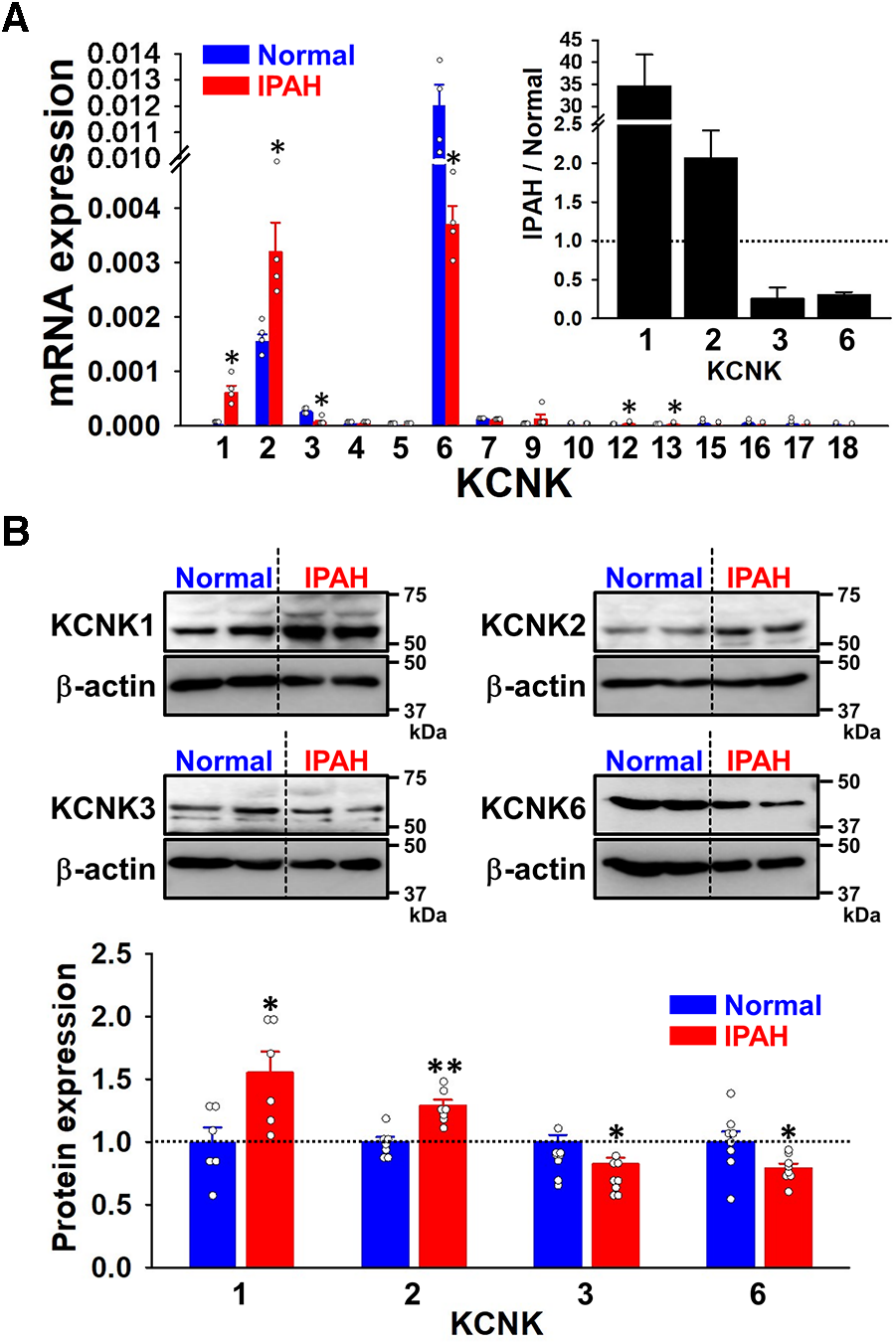
Expression profiles of KCNK family members in PASMCs from IPAH patients. The expression of KCNK channel family members (KCNK1 to 18, except for 8, 11, and 14) in normal- and IPAH-PASMCs was examined by qPCR and Western blotting. (**A**) Expression of KCNK family members in normal- and IPAH-PASMCs at the mRNA level (*n* = 4). The mRNA expression level of KCNK was normalized to that of β-actin. *Inset*, the expression ratios of KCNK1, 2, 3, and 6 in IPAH-PASMCs to normal-PASMCs (*n* = 4). (**B**) Protein expression of KCNK1, 2, 3, and 6 channels in normal- and IPAH-PASMCs (*n* = 6–8). The protein expression of KCNK channels was normalized to that of β-actin and normal-PASMCs. Note that the expression of KCNK1/TWIK1 and KCNK2/TREK1 was up-regulated, whereas that of KCNK3/TASK1 and KCNK6/TWIK2 was down-regulated in IPAH-PASMCs. Data are presented as means ± S.E. *p < 0.05, **p < 0.01 vs. normal-PASMCs (Mann–Whitney *U* test).

The expression of KCNK channel genes (KCNK1 to 18, except for 8, 11, and 14) was analyzed in PASMCs from normal subjects and IPAH patients by qPCR. KCNK2/TREK1, KCNK3/TASK1, and KCNK6/TWIK2 genes were detected in normal-PASMCs (Figure 1A and Supplementary Table S1). The mRNA expression of KCNK1/TWIK1 (0.00061 ± 0.00012 of β-actin, *n* = 4, *p* = 0.029 vs. normal, 0.00002 ± 0.00001, *n* = 4; IPAH/normal ratio = 34.70 ± 7.05-fold) and KCNK2 (0.00319 ± 0.00054, *n* = 4, *p* = 0.029 vs. normal, 0.00154 ± 0.00014, *n* = 4; 2.07 ± 0.35-fold) channels was up-regulated in IPAH-PASMCs. In contrast, the mRNA expression of KCNK3 (0.00006 ± 0.00004, *n* = 4, *p* = 0.029 vs. normal, 0.00025 ± 0.00003, *n* = 4; 0.26 ± 0.14-fold) and KCNK6 (0.00366 ± 0.00034, *n* = 4, *p* = 0.029 vs. normal, 0.01185 ± 0.00083, *n* = 4; 0.31 ± 0.03-fold) channels was down-regulated in IPAH-PASMCs.

The expression levels of the KCNK1, 2, 3, and 6 proteins in normal- and IPAH-PASMCs were compared by Western blotting. The protein expression of KCNK1 channels was higher in IPAH-PASMCs than in normal-PASMCs (1.51 ± 0.17-fold, *n* = 6, *p* = 0.041 vs. normal, 1.00 ± 0.14, *n* = 6) (Figure 1B). The protein expression of KCNK2 was also higher in IPAH-PASMCs (1.29 ± 0.05-fold, *n* = 7, *p* = 0.002 vs. normal, 1.00 ± 0.04, *n* = 7). In contrast, the protein expression levels of KCNK3 and KCNK6 were lower in IPAH-PASMCs than in normal-PASMCs (0.82 ± 0.05-fold, *n* = 8, *p* = 0.015 and 0.79 ± 0.04-fold, *n* = 8, *p* = 0.028, respectively). These results indicated that the expression of KCNK1 and KCNK2 channels was up-regulated, whereas that of KCNK3 and KCNK6 channels was down-regulated in PASMCs from IPAH patients.

### 3.2 Changes in KCNK1 and KCNK2 channel expression in experimental PH animals

**Figure 2:**
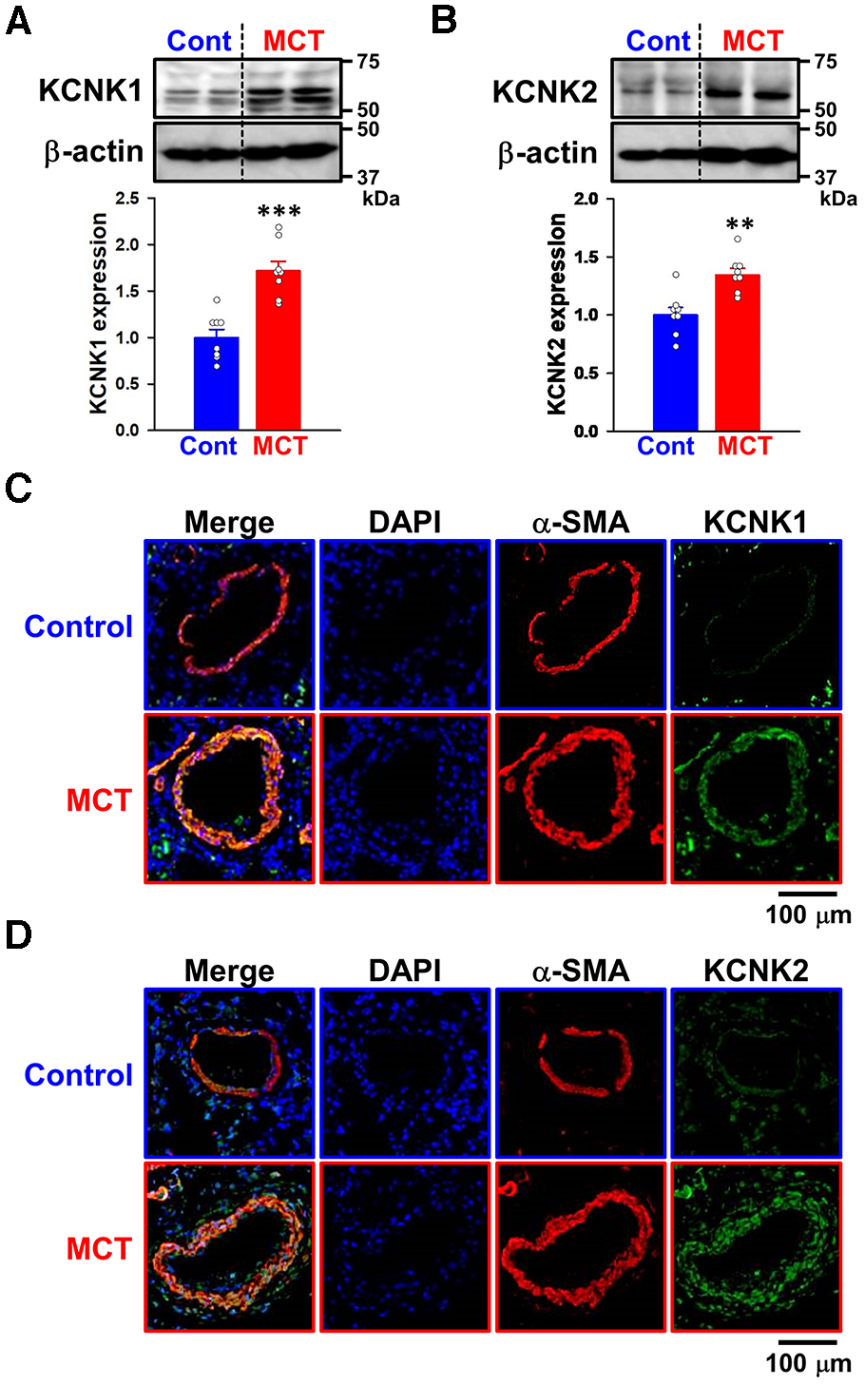
Up-regulated expression of KCNK1 and KCNK2 channels in PASMs from MCT-PH rats. The protein expression of KCNK1 and KCNK2 channels in PASMs from MCT-PH rats was examined by Western blotting and immunohistochemical staining. (**A,B**) Protein expression of KCNK1 (**A**) and KCNK2 (**B**) channels in PASMCs from control and MCT-PH rats (*n* = 8). Protein expression was normalized to that of β-actin and the control group. (**C,D**) Representative immunohistochemical images of the lung sections of control and MCT-PH rats stained with a KCNK1 (**C**; *green*), KCNK2 (**D**; *green*), or *α*-SMA (*red*) antibody. Cell nuclei were stained with DAPI (*blue*). Similar results were obtained from six independent experiments. Note that the expression of KCNK1/TWIK1 and KCNK2/TREK1 was up-regulated in PASMs from MCT-PH rats. Data are presented as means ± S.E. **p < 0.01, ***p < 0.001 vs. the control (Mann–Whitney *U* test).

Since the expression of KCNK1 and KCNK2 channels was up-regulated in IPAH-PASMCs, their expression changes in PASMs from three types of experimental PH animals (MCT-PH rats, SuHx-PH rats, and hypoxia-PH mice) were also examined. It was confirmed that endothelium was not attached to the dissected PASMs because endothelium-dependent relaxation was not induced by acetylcholine (25). The expression of KCNK1 and KCNK2 channel proteins was examined in PASMs from control and MCT-PH rats by Western blotting. The protein expression of KCNK1 (1.72 ± 0.10-fold, *n* = 8, *p* < 0.001 vs. control, 1.00 ± 0.09, *n* = 8) and KCNK2 (1.35 ± 0.06-fold, *n* = 8, *p* = 0.002 vs. control, 1.00 ± 0.06, *n* = 8) channels was increased in MCT-PASMs (Figures 2A,B). In addition, the expression of KCNK1 and KCNK2 channel proteins was analyzed using the lung sections of control and MCT-PH rats by immunohistochemical staining. Immunohistochemical images revealed that KCNK1 and KCNK2 channels were localized in the medial (smooth muscle) layer of the pulmonary artery and their expression was higher in MCT-PH rats than in the control rats (Figures 2C,D).

**Figure 3:**
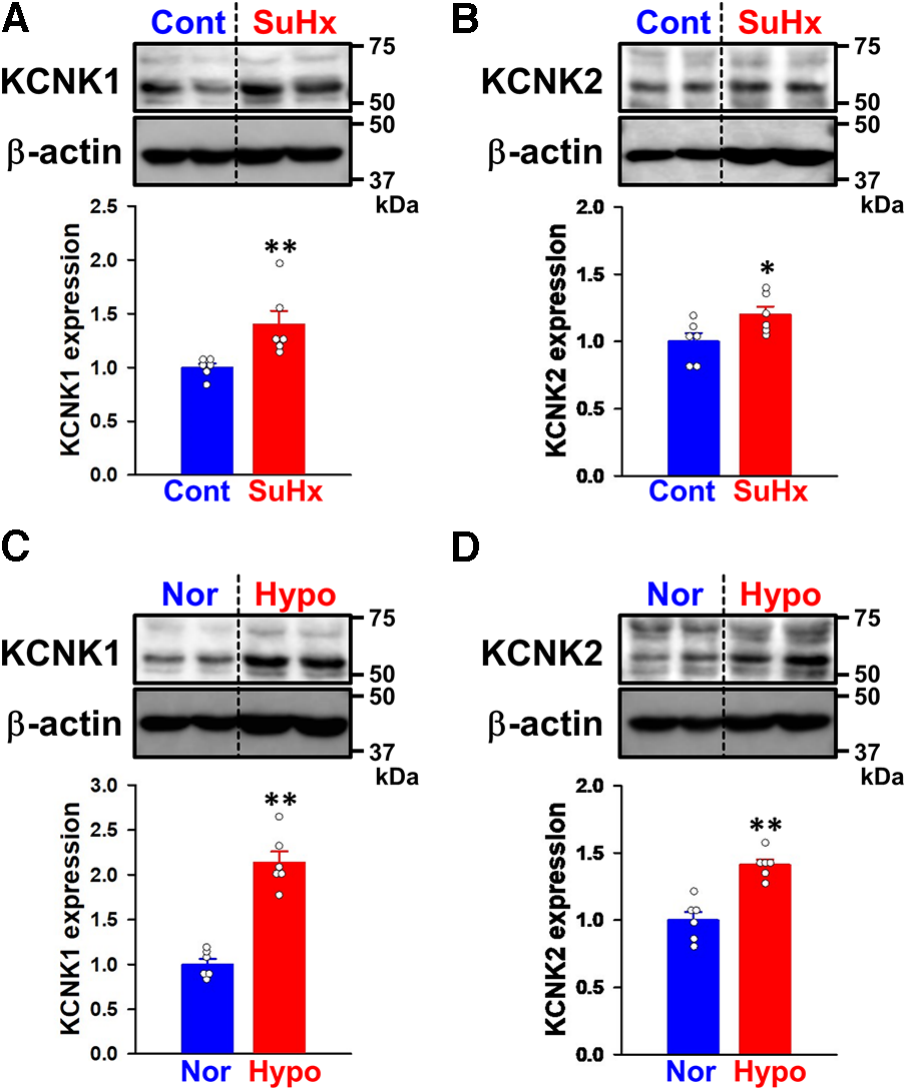
Expression of KCNK1 and KCNK2 channels in PASMs from SuHx-PH rats and hypoxia-PH mice. The protein expression of KCNK1 and KCNK2 channels in PASMs from SuHx-PH rats and hypoxia-PH mice was examined by Western blotting. (**A,B**) Protein expression of KCNK1 (**A**) and KCNK2 (**B**) channels in PASMs from control and SuHx-PH rats (*n* = 6). Protein expression was normalized to that of β-actin and the control group. (**C,D**) Protein expression of KCNK1 (**C**) and KCNK2 (**D**) channels in PASMs from normoxia and hypoxia-PH mice (*n* = 6). Protein expression was normalized to that of β-actin and the normoxia group. Data are presented as means ± S.E. *p < 0.05, **p < 0.01 vs. the control or normoxia group (Mann–Whitney *U* test).

Changes in KCNK1 and KCNK2 protein expression were then assessed in PASMs from control and SuHx-PH rats by Western blotting. The expression of KCNK1 (1.40 ± 0.13-fold, *n* = 6, *p* = 0.002 vs. control, 1.00 ± 0.04, *n* = 6) and KCNK2 (1.20 ± 0.06, *n* = 6, *p* = 0.041 vs. control, 1.00 ± 0.06, *n* = 6) channels was up-regulated in PASMs from SuHx-PH rats (Figures 3A,B).

KCNK1 and KCNK2 protein expression levels were also evaluated in PASMs from normoxia and hypoxia-PH mice. The expression of KCNK1 (2.14 ± 0.12-fold, *n* = 6, *p* = 0.002 vs. normoxia, 1.00 ± 0.06, *n* = 6) and KCNK2 (1.41 ± 0.04-fold, *n* = 6, *p* = 0.002 vs. normoxia, 1.00 ± 0.06, *n* = 6) channels was increased in PASMs from hypoxia-PH mice (Figures 3C,D). These results strongly suggest the up-regulated expression of KCNK1 and KCNK2 channels in PASMs from three types of experimental PH animals, similar to PAH patients.

### 3.3 Inhibitory effects of KCNK channel blockers on the excessive proliferation of IPAH-PASMCs

**Figure 4:**
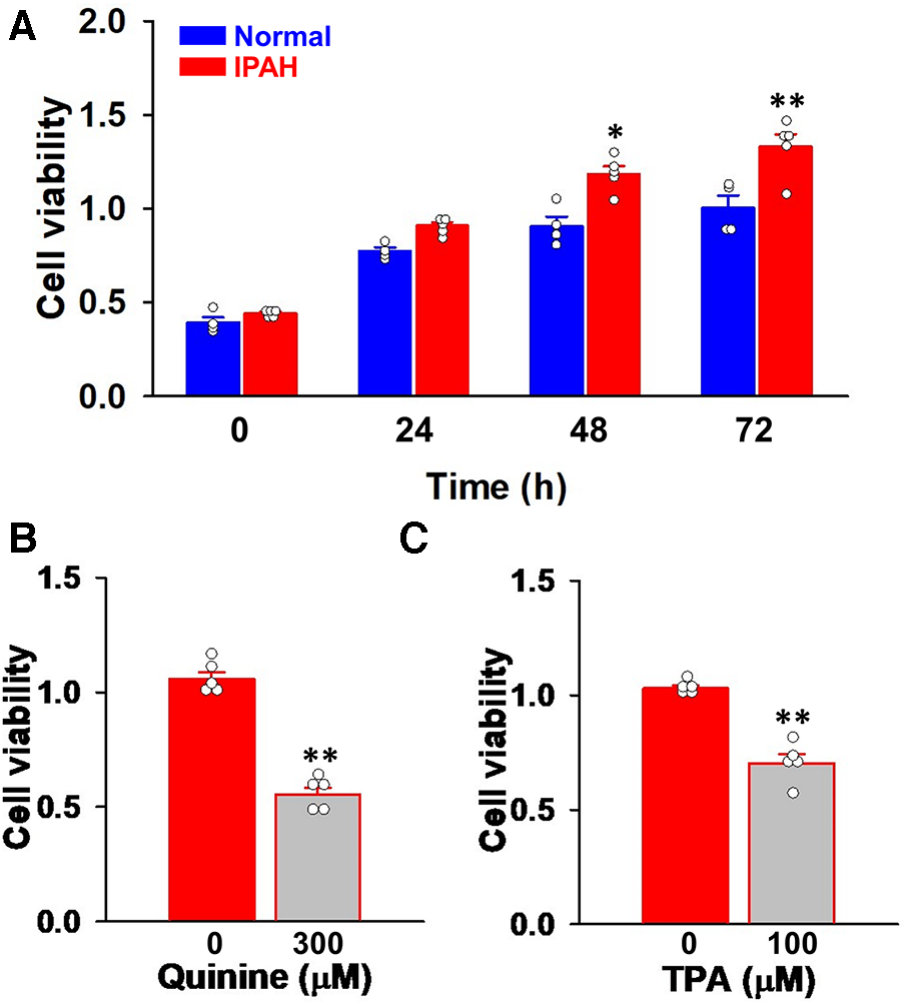
Effects of KCNK channel blockers on the excessive proliferation of IPAH-PASMCs. The effects of the KCNK channel blockers, quinine and TPA, on the proliferation of IPAH-PASMCs were examined using the Cell Counting Kit-8 assay. (**A**) The growth of normal- and IPAH-PASMCs after a 24-, 48-, or 72-h culture (*n* = 4). (**B**) Effects of the treatment with 300 μM quinine for 48 h on the excessive proliferation of IPAH-PASMCs (*n* = 5). (**C**) Effects of 100 μM TPA for 48 h on the excessive proliferation of IPAH-PASMCs (*n* = 5). Data are presented as means ± S.E. *p < 0.05, **p < 0.01 vs. normal-PASMCs or the control (Scheffé's test following Kruskal–Wallis test (**A**) or Mann–Whitney *U* test (**B,C**)).

Since vascular remodeling in PAH is predominantly mediated by the excessive proliferation of PASMCs (4, 5), the involvement of KCNK channels was examined using Cell Counting Kit-8. Cell viability increases in a time-dependent manner in both normal-PASMCs (A_450_ = 0.393 ± 0.028 at 0 h, 0.775 ± 0.020 at 24 h, 0.906 ± 0.052 at 48 h, and 1.004 ± 0.067 at 72 h, *n* = 4) and IPAH-PASMCs (0.441 ± 0.008 at 0 h, 0.909 ± 0.019 at 24 h, 1.184 ± 0.042 at 48 h, and 1.330 ± 0.067 at 72 h, *n* = 5) (Figure 4A). The growth of IPAH-PASMCs at 48 and 72 h was markedly greater than that of normal-PASMCs (*p* = 0.020 and *p* = 0.004, respectively), which is consistent with previous findings (23, 26, 27).

The effects of KCNK channel blockers on the facilitated proliferation of IPAH-PASMCs were examined. In IPAH-PASMCs, increases in proliferation were reduced by a treatment with 300 μM quinine, a KCNK channel blocker (10, 28, 29), for 48 h (A_450_ = 0.552 ± 0.032, *n* = 5, *p* = 0.008 vs. control, 1.057 ± 0.032, *n* = 5) (Figure 4B). A similar reduction was observed with 100 μM TPA, another KCNK channel blocker that is structurally different from quinine (29–32), for 48 h (0.703 ± 0.039, *n* = 5, *p* = 0.008 vs. control, 1.030 ± 0.013, *n* = 5) (Figure 4C). This result suggests that up-regulated KCNK channel function involved in the enhanced proliferation of IPAH-PASMCs.

### 3.4 Anti-migratory effects of KCNK channel blockers in IPAH-PASMCs

**Figure 5:**
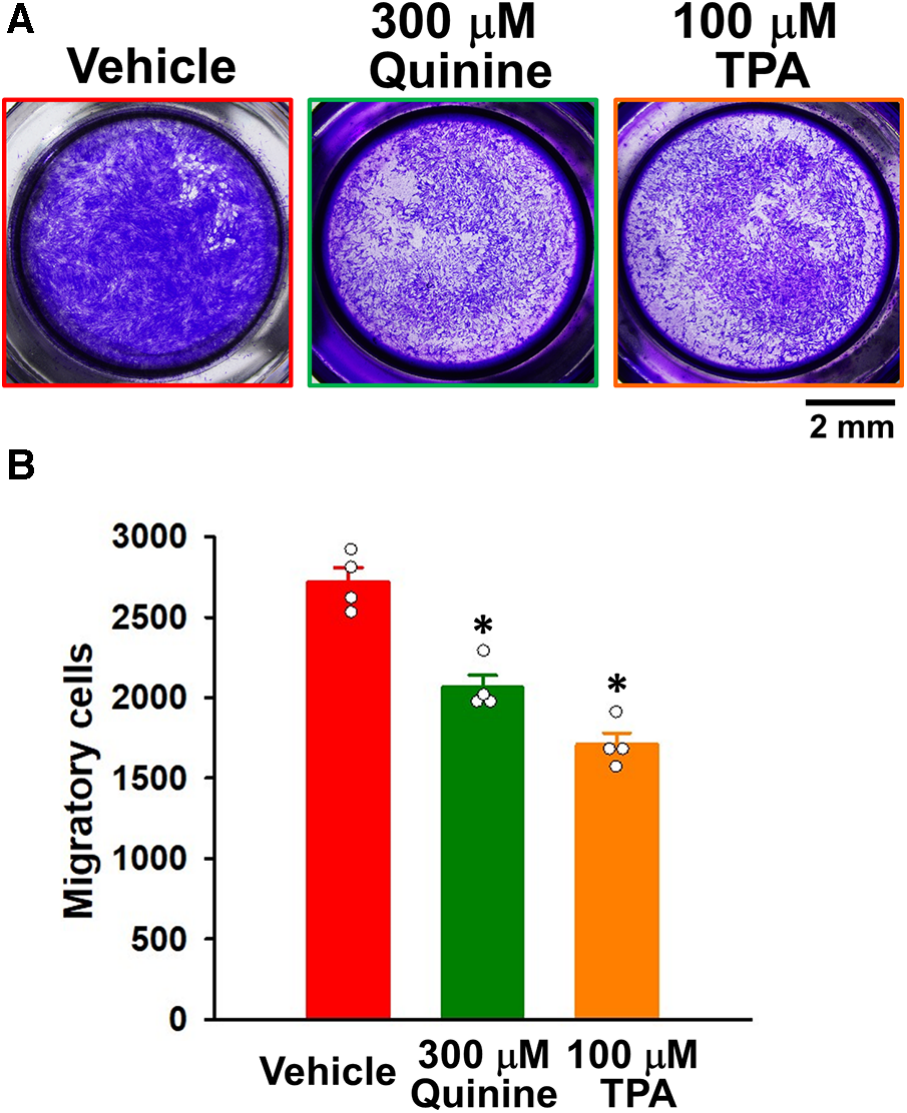
Effects of KCNK channel blockers on the migration of IPAH-PASMCs. The effects of the KCNK channel blockers, quinine and TPA, on the migration of IPAH-PASMCs were examined using the Transwell assay. (**A**) Representative images of migrated PASMCs stained with crystal violet after the treatment with vehicle, 300 μM quinine, or 100 μM TPA for 24 h. (**B**) Effects of quinine and TPA for 24 h on the migration of IPAH-PASMCs (*n* = 4). Data are presented as means ± S.E. *p < 0.05 vs. the vehicle (Steel's test following Kruskal–Wallis test).

The effects of KCNK channel blockers on the migration of IPAH-PASMCs were investigated by Transwell assays. The migration was inhibited by 300 μM quinine for 24 h (2,065 ± 76 cells, *n* = 4, *p* = 0.039 vs. control, 2,720 ± 88 cells, *n* = 4) (Figure 5). It was also reduced by 100 μM TPA for 24 h (1,710 ± 72 cells, *n* = 4, *p* = 0.039). Collectively, these results suggest that up-regulated KCNK channels mediated the migration and excessive proliferation of IPAH-PASMCs.

### 3.5 Involvement of KCNK1 and KCNK2 channels in the proliferation and migration of IPAH-PASMCs

**Figure 6:**
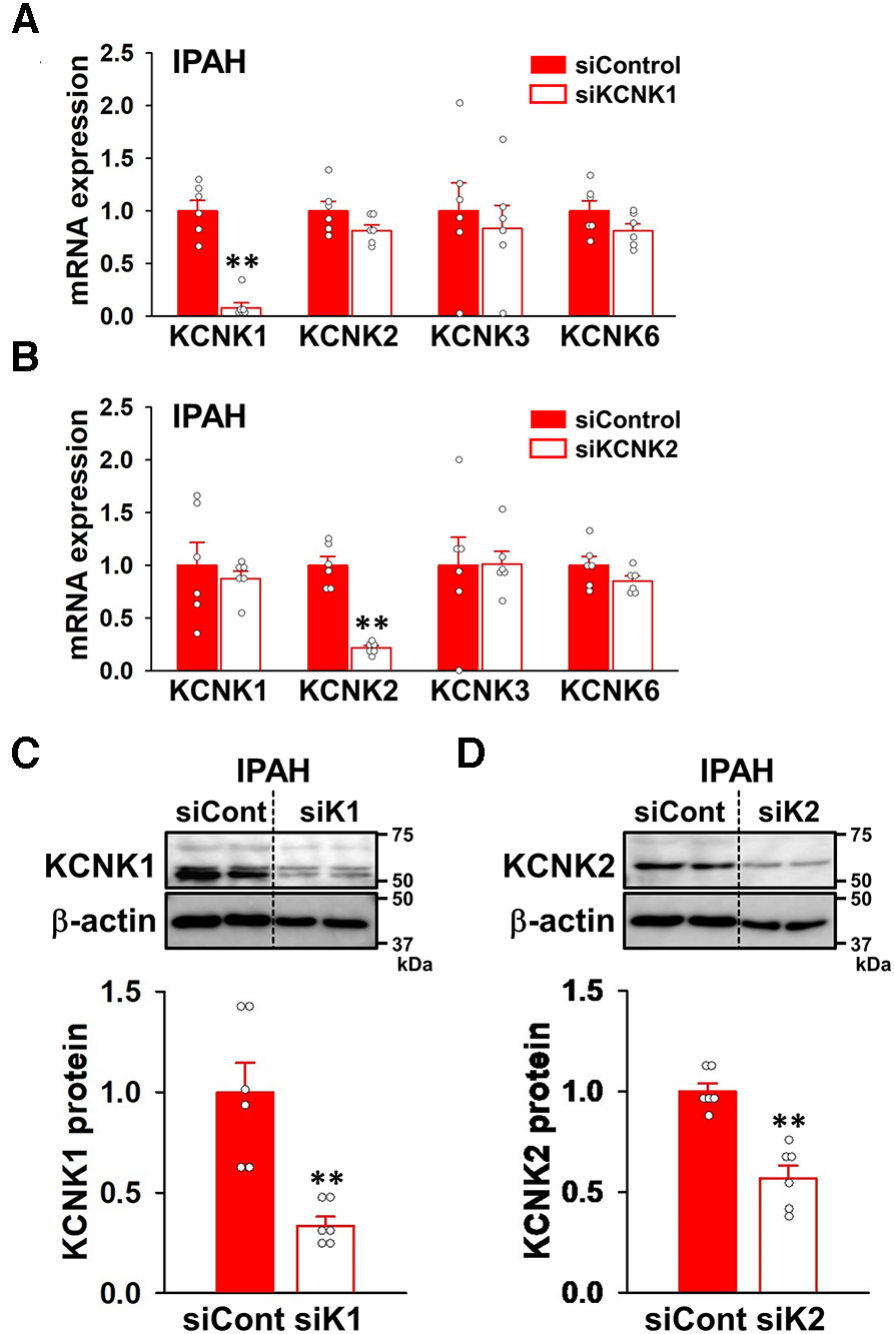
siRNA knockdown of KCNK1 and KCNK2 channels in IPAH-PASMCs. The effects of KCNK1 and KCNK2 siRNAs on the expression of KCNK1, KCNK2, KCNK3, and KCNK6 channels in IPAH-PASMCs were examined by qPCR and Western blotting. (**A,B**) Knockdown efficiency at the mRNA level of siRNA targeting KCNK1 (**A**) or KCNK2 (**B**) in IPAH-PASMCs (*n* = 6). mRNA expression was normalized to that of β-actin and control siRNA. (**C,D**) Knockdown efficiency at the protein level of siRNA targeting KCNK1 (**C**) or KCNK2 (**D**) in IPAH-PASMCs (*n* = 6). Protein expression was normalized to that of β-actin and control siRNA. Data are presented as means ± S.E. **p < 0.01 vs. control siRNA (Mann–Whitney *U* test).

To obtain direct evidence for the contribution of KCNK1 and KCNK2 channels in the proliferation of IPAH-PASMCs, cell viability and proliferation were examined by WST-8 and BrdU incorporation assays after KCNK1 or KCNK2 specific knockdown by siRNAs. The knockdown efficacy by KCNK1 and KCNK2 siRNA was confirmed by qPCR and Western blotting. KCNK1 siRNA knocked-down the mRNA expression of KCNK1 channels in IPAH-PASMCs (92.4 ± 5.0% decrease, *n* = 6, *p* = 0.002 vs. control siRNA, *n* = 6), whereas it did not affect the expression levels of KCNK2, KCNK3, or KCNK6 channels (*n* = 6, *p* > 0.05) (Figure 6A). KCNK2 siRNA knocked-down KCNK2 expression (78.1 ± 2.2% decrease, *n* = 6, *p* = 0.002 vs. control siRNA, *n* = 6), but did not affect the expression of KCNK1, KCNK3, or KCNK6 channels (*n* = 6, *p* > 0.05) (Figure 6B). Similarly, KCNK1 siRNA knocked-down KCNK1 proteins (66.3 ± 4.4% decrease, *n* = 6, *p* = 0.002) (Figure 6C) and KCNK2 siRNA knocked-down KCNK2 proteins (43.0 ± 6.3% decrease, *n* = 6, *p* = 0.002) (Figure 6D) in IPAH-PASMCs.

**Figure 7:**
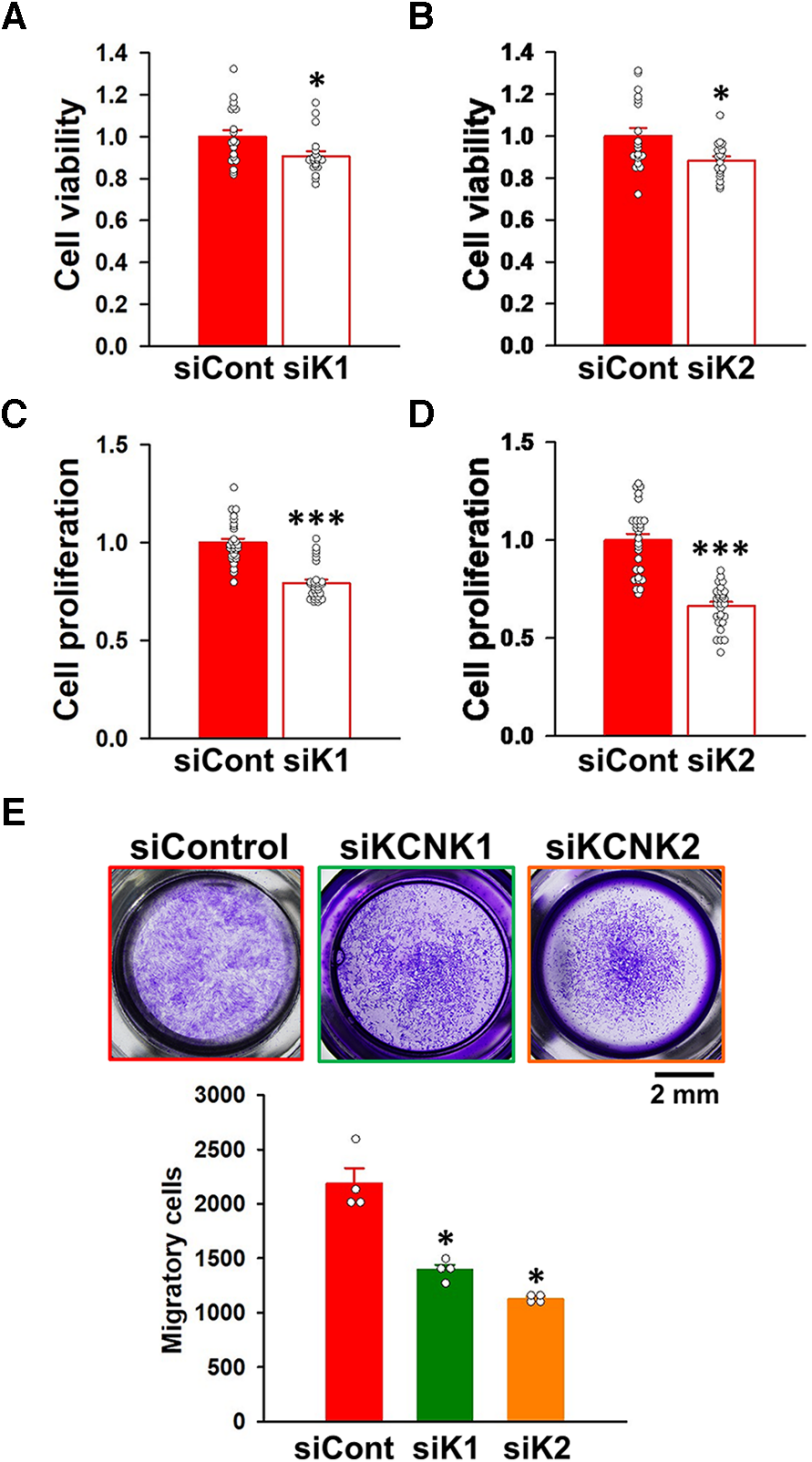
Contribution of KCNK1 and KCNK2 channels to the proliferation and migration of IPAH-PASMCs. The involvement of KCNK1 and KCNK2 channels in the proliferation and migration of IPAH-PASMCs was examined by siRNA knockdown methods. (**A,B**) Inhibitory effects of the transfection with KCNK1 (**A**) or KCNK2 (**B**) siRNA for 48 h on the growth of IPAH-PASMCs using the Cell Counting Kit-8 assay (*n* = 4). Absorbance was normalized by control siRNA. (**C,D**) Inhibitory effects of the transfection with KCNK1 (**C**) or KCNK2 (**D**) siRNA for 48 h on the excessive proliferation of IPAH-PASMCs using the BrdU incorporation assay (*n* = 4). Absorbance was normalized by control siRNA. (**E**) Anti-migratory effects of the transfection with KCNK1 or KCNK2 siRNA on the migration of IPAH-PASMCs for 24 h using the Transwell assay (*n* = 4). Data are presented as means ± S.E. *p < 0.05, **p < 0.001 vs. control siRNA (Student's *t*-test (**A–D**) or Steel's test following Kruskal-Wallis test (**E**)).

When KCNK1 siRNA was transfected into IPAH-PASMCs for 48 h, their excessive proliferation was significantly attenuated (9.3 ± 2.3% decrease, *n* = 19, *p* = 0.025 vs. control siRNA, *n* = 19) (Figure 7A). KCNK2 siRNA for 48 h also suppressed the facilitated proliferation (11.6 ± 2.0% decrease, *n* = 19, *p* = 0.013 vs. control siRNA, *n* = 19) (Figure 7B). To confirm the results obtained from the WST-8 assay, the BrdU assay was performed on IPAH-PASMCs. The excessive proliferation was inhibited by the siRNA knockdown of KCNK1 (20.5 ± 1.8% decrease, *n* = 28, *p* < 0.001 vs. control siRNA, *n* = 28) or KCNK2 (33.5 ± 2.0% decrease, *n* = 28, *p* < 0.001 vs. control siRNA, *n* = 28) for 48 h (Figures 7C,D).

The effects of KCNK1 and KCNK2 siRNA on the migration of IPAH-PASMCs were assayed using the Transwell plates. KCNK1 siRNA inhibited the migration of IPAH-PASMCs for 24 h (1,394 ± 47 cells, *n* = 4, *p* = 0.039 vs. control siRNA, 2,189 ± 139 cells, *n* = 4) (Figure 7E). KCNK2 siRNA also suppressed the migration of IPAH-PASMCs for 24 h (1,130 ± 20 cells, *n* = 4, *p* = 0.039). Collectively, these findings strongly indicate that the activities of KCNK1 and KCNK2 channels were responsible for the proliferation and migration of IPAH-PASMCs, leading to vascular remodeling in PAH.

### 3.6 Contribution of KCNK1 and KCNK2 channels to the resting membrane potential and [Ca^2+^]_cyt_ in IPAH-PASMCs

**Figure 8:**
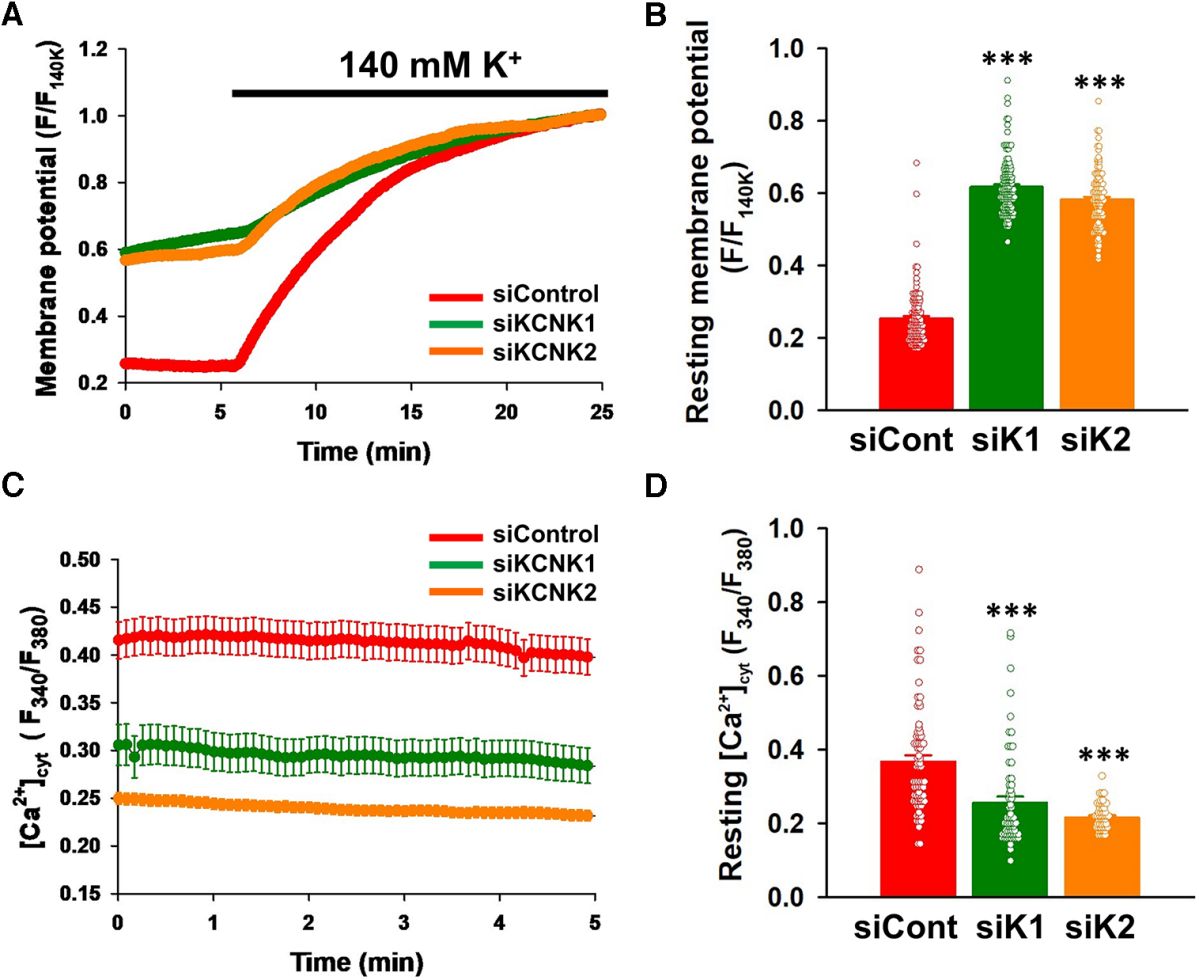
Contribution of KCNK1 and KCNK2 channels to the resting membrane potential and [Ca^2+^]_cyt_ in IPAH-PASMCs. The effects of the siRNA knockdown of KCNK1 and KCNK2 on the resting membrane potential and [Ca^2+^]_cyt_ were measured in IPAH-PASMCs. Membrane potential was monitored with the voltage-sensitive fluorescent indicator, DiBAC_4_(3). Fluorescent intensity of DiBAC_4_(3) (F/F_140K_) was increased and decreased by membrane depolarization and hyperpolarization, respectively. Fluorescent intensity signal was normalized by the maximum fluorescent intensity in the 140-mM K^+^ HEPES-buffered solution (theoretically 0 mV). [Ca^2+^]_cyt_ (F_340_/F_380_) was measured using the Ca^2+^-sensitive fluorescent indicator, fura-2/AM. (**A**) Time courses of the membrane potential in IPAH-PASMCs transfected with control (*n* = 115), KCNK1 (*n* = 131), or KCNK2 (*n* = 98) siRNA before and after the perfusion with 140 mM K^+^ HEPES-buffered solution. (**B**) Summarized data of the resting membrane potential in control (*n* = 115), KCNK1 (*n* = 131), or KCNK2 (*n* = 98) siRNA-treated IPAH-PASMCs. The resting membrane potential was defined as the average value of F/F_140K_ for 5 min before the perfusion with 140 mM K^+^ HEPES-buffered solution. (**C**) Resting [Ca^2+^]_cyt_ levels in IPAH-PASMCs transfected with control (*n* = 73), KCNK1 (*n* = 63), or KCNK2 (*n* = 48) siRNA. (**D**) Summarized data of the resting [Ca^2+^]_cyt_ in control (*n* = 73), KCNK1 (*n* = 63), or KCNK2 (*n* = 48) siRNA-treated IPAH-PASMCs. The resting [Ca^2+^]_cyt_ was defined as the average value of F_340_/F_380_ for 5 min after the beginning of the experiment. Data are presented as means ± S.E. ***p < 0.001 vs. control siRNA (Scheffé's test following ANOVA).

We examined whether the activities of KCNK1 and KCNK2 channels contributed to the resting membrane potential of IPAH-PASMCs by fluorescence imaging using the voltage-sensitive dye, DiBAC_4_(3). In IPAH-PASMCs, the resting membrane potential was shifted in the depolarizing direction by the siRNA knockdown of KCNK1 channels (F/F_140K_ = 0.617 ± 0.007, *n* = 131, *p* < 0.001 vs. control siRNA, 0.253 ± 0.007, *n* = 115) (Figures 8A,B). Similar depolarizing changes in the resting membrane potential were induced by the siRNA knockdown of KCNK2 channels (0.582 ± 0.008, *n* = 98, *p* < 0.001).

Since membrane depolarization decreases [Ca^2+^]_cyt_ in a proliferative phenotype of vascular myocytes (33, 34), the effects of the siRNA knockdown of KCNK1 and KCNK2 channels on resting [Ca^2+^]_cyt_ in IPAH-PASMCs were examined by fluorescence imaging using the Ca^2+^ indicator, fura-2/AM. KCNK1 siRNA-treated IPAH-PASMCs showed lower resting [Ca^2+^]_cyt_ than control siRNA-treated cells (F_340_/F_380_ = 0.255 ± 0.017, *n* = 63, *p* < 0.001 vs. control siRNA, 0.367 ± 0.018, *n* = 73) (Figures 8C,D). KCNK2 siRNA-treated cells also had a lower resting [Ca^2+^]_cyt_ than control cells (0.216 ± 0.005, *n* = 48, *p* < 0.001). Taken together, these findings suggest that up-regulated KCNK1 and KCNK2 channel expression caused membrane hyperpolarization and subsequent [Ca^2+^]_cyt_ increases in IPAH-PASMCs.

### 3.7 Regulation of the phosphorylation of JNK by KCNK1 and KCNK2 channels in IPAH-PASMCs

**Figure 9:**
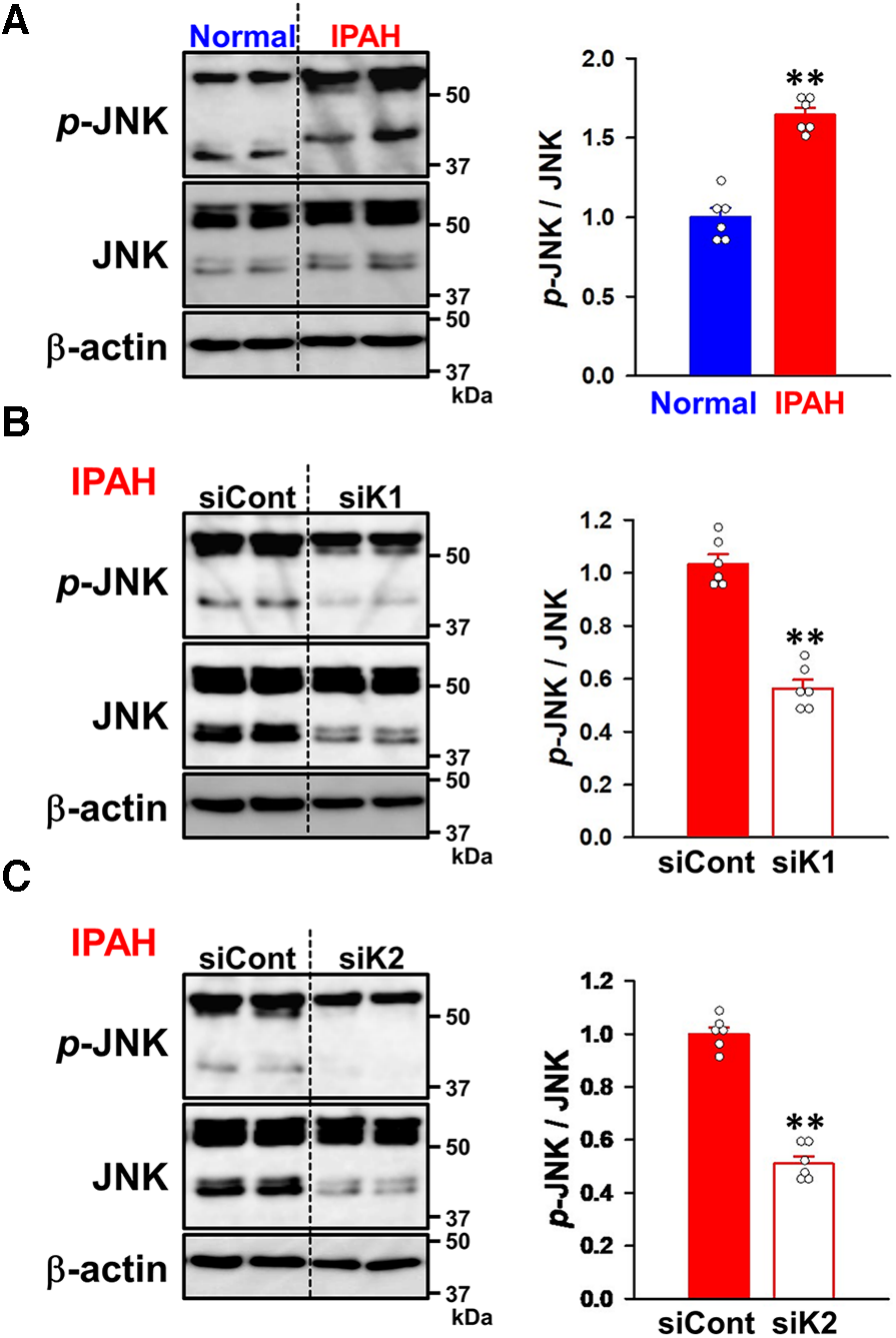
Effects of KCNK1 and KCNK2 channel knockdown on the phosphorylation of JNK in IPAH-PASMCs. The expression and phosphorylation levels of JNK in normal- and IPAH-PASMCs and the effects of the siRNA knockdown of KCNK1 and KCNK2 channels were examined by Western blotting. (**A**) The phosphorylation levels of JNK in normal- and IPAH-PASMCs (*n* = 6). Protein expression was normalized to that of β-actin and normal-PASMCs. (**B**) The effects of the siRNA knockdown of KCNK1 channels on the phosphorylation levels of JNK in IPAH-PASMCs (*n* = 6). Protein expression was normalized to that of β-actin and control siRNA. (**C**) The effects of the siRNA knockdown of KCNK2 channels on the phosphorylation levels of JNK in IPAH-PASMCs (*n* = 6). Protein expression was normalized to that of β-actin and control siRNA. Data are presented as means ± S.E. **p < 0.01, **p < 0.01 vs. normal-PASMCs or control siRNA (Mann–Whitney *U* test).

To elucidate the involvement of KCNK1 and KCNK2 channels in the proliferation and migration of IPAH-PASMCs, we focused on the JNK signaling pathway, which is involved in vascular remodeling in PAH (12–17). Western blot analyses revealed that the phosphorylation level of JNK was facilitated in IPAH-PASMCs compared to in normal-PASMCs (1.65 ± 0.04-fold, *n* = 6, *p* = 0.002 vs. normal, 1.00 ± 0.06, *n* = 6) (Figure 9A). Up-regulated JNK phosphorylation in IPAH-PASMCs was decreased by the siRNA knockdown of KCNK1 channels (43.7 ± 3.3% decrease, *n* = 6, *p* = 0.002) (Figure 9B). Similarly, it was down-regulated by the siRNA knockdown of KCNK2 channels (48.9 ± 2.7% decrease, *n* = 6, *p* = 0.002) (Figure 9C). These findings suggest that the expression and activity of KCNK1 and KCNK2 channels affected the phosphorylation levels of JNK in IPAH-PASMCs, thereby facilitating the proliferation and migration of PASMCs in PAH.

## 4 Discussion

The present investigation demonstrated that the expression of KCNK1/TWIK1 and KCNK2/TREK1 channels was up-regulated in PASMCs from IPAH patients and experimental PH animals and their up-regulation facilitated the proliferation and migration of IPAH-PASMCs via enhanced Ca^2+^ signaling and JNK signaling pathway, resulting in vascular remodeling in PAH.

In vascular myocytes, including PASMCs, [Ca^2+^]_cyt_ increment is required for cellular contraction, proliferation, migration, apoptosis, and the cell cycle. [Ca^2+^]_cyt_ is modulated by the resting membrane potential, which is mainly affected by K^+^ channel conductance. Therefore, K^+^ channels are recognized as an important molecule for the modulation of cytosolic Ca^2+^ mobilization (4, 5). KCNK channels are responsible for background or leak K^+^ currents, which maintain the resting membrane potential (8, 35). Among members of the KCNK channel family, the expression of the KCNK2, 3, 5, and 6 channels was detected in PASMCs (6, 7, 36). Specifically, loss-of-function mutations in KCNK3 channels have been implicated in heritable PAH (3, 37, 38). Furthermore, the expression of KCNK3 channels was down-regulated in PASMCs treated with hypoxia (39), from IPAH patients and MCT-PH rats (40), and KCNK3-knockdown rats exhibit PH (41). On the other hand, KCNK6-knockout mice developed PH (42), whereas the expression of KCNK6 channels was unchanged in IPAH patients (7). In the present study, KCNK3/TASK1 (29.8 and 30.2% homology with KCNK1 and KCNK2, respectively) and KCNK6/TWIK2 (46.9 and 33.1%) channel expression at the protein level was slightly down-regulated by 18% and 21%, respectively, in PASMCs from IPAH patients. The most interesting finding of this investigation is that KCNK1/TWIK1 and KCNK2/TREK1 (33.9% homology to each other) channel expression at the protein level was up-regulated to 151% and 129%, respectively, in IPAH-PASMCs. Similar up-regulation was observed in PASMs from MCT-PH rats, SuHx-PH rats, and hypoxia-PH mice. These findings suppose that the mechanism responsible for this up-regulation is common between IPAH patients and experimental PH animals. In the right ventricle from MCT-PH rats, the mRNA expression of KCNK2 and KCNK3 channels was up-regulated and down-regulated, respectively (Supplementary Table S2). The expression of KCNK1 and KCNK6 channels was unchanged between control and MCT-PH rats. The mRNA expression of KCNK3 channels has been reported to be down-regulated in the right ventricle from PAH patients and experimental PH rats (43–45). To the best of our knowledge, this is the first study to comprehensively demonstrate changes in KCNK channel expression in PASMCs from IPAH patients. These up-regulated and down-regulated expression may be a compensatory mechanism for each other. Therefore, further experiments are required regarding the functional relationship between these expression changes.

An increase in [Ca^2+^]_cyt_ at physiological ranges triggers the proliferation and migration of PASMCs, however, [Ca^2+^]_cyt_ overload also facilitate the proliferation and migration of PASMCs and subsequent pulmonary vascular remodeling, resulting in the development and progression of PAH (4, 5). In the present investigation, the KCNK channel blockers, quinine and TPA, blocked the proliferation and migration of IPAH-PASMCs. Previous studies reported that quinine blocked the KCNK1 and KCNK2 (also KCNK5, 6, 9, 16, and 18) channels (10, 28, 29), while TPA blocked the KCNK1 and KCNK2 (also KCNK4, 9, 10, 17, and 18) channels (29–32). These blockers were slightly less selective, but still inhibited the activities of KCNK1 and KCNK2 channels. Therefore, the effects of these blockers on the proliferation and migration of IPAH-PASMCs appear to be mediated by the suppression of up-regulated KCNK1 and KCNK2 channels. The results of siRNA knockdown experiments strongly suggest the involvement of KCNK1 and KCNK2 channel activities in the enhanced proliferation and migration of IPAH-PASMCs following membrane hyperpolarization and [Ca^2+^]_cyt_ increase. The increased activity of K^+^ channels induces membrane hyperpolarization. Since IPAH-PASMCs exhibit a proliferative or synthetic phenotype, Ca^2+^ influx is largely mediated by voltage-independent Ca^2+^ channels (e.g., ROC and SOC channels), but not by VDCCs (33). Therefore, membrane hyperpolarization due to the up-regulation of KCNK1/KCNK2 channel expression facilitates Ca^2+^ influx through ROC and SOC channels in IPAH-PASMCs, similar to that in non-excitable cells, such as epithelial, endothelial, immune, and cancer cells (34). Enhanced Ca^2+^ signaling contributes to the facilitated proliferation and migration of IPAH-PASMCs, leading to pulmonary vascular remodeling and the progression of PAH.

We found that the expression of Ca^2+^-permeable/sensitive channels and receptors, transient receptor potential (TRP) canonical channels (TRPC6) (24, 46, 47), TRP vanilloid channels (TRPV1 and TRPV4) (48, 49), TRP melastatin channels (TRPM7) (48), Orai/STIM channels (Orai1, Orai2, and STIM2) (46), and Ca^2+^-sensing receptors (26, 50, 51), was up-regulated, and thus, involved in abnormal Ca^2+^ events in PAH. We recently demonstrated that the expression of large-conductance Ca^2+^-activated K^+^ channels (K_Ca_1.1) was down-regulated in IPAH-PASMCs (52), whereas that of swelling-activated Cl^−^ channels (ClC-3) was up-regulated in IPAH-PASMCs (27). In addition, the present investigation clearly showed the involvement of up-regulated KCNK1/TWIK1 and KCNK2/TREK1 channels in the vascular remodeling of PAH.

Since the activation of JNK signaling, belonging to the MAPK family (11), was identified as one of the mechanisms underlying vascular remodeling in experimental PH animals (12, 13, 16, 17) and PAH patients (14), we focused on the JNK signaling pathway in the present study. In addition, KCNK2 channels are necessary for JNK activation in response to pressure overload in cardiomyocytes and fibroblasts, which leads to cardiac remodeling (53). The phosphorylation of JNK was enhanced in IPAH-PASMCs compared to in normal-PASMCs (165%), which is consistent with previous findings (14). The facilitated phosphorylation was markedly suppressed by the knockdown of KCNK1 or KCNK2 channels, suggesting that the activity and/or expression of KCNK1/KCNK2 channels contribute to the phosphorylation of JNK signaling pathway in IPAH-PASMCs. The up-regulated expression of KCNK1/KCNK2 channels has been suggested to shift the resting membrane potential in a hyperpolarizing direction, enhance Ca^2+^ influx, and facilitate Ca^2+^-dependent signaling, including JNK (5). In addition to vascular remodeling, JNK signaling is suggested to contribute to the process of inflammation (11), which is one of the pathological hallmarks of PAH (1). Some KCNK channels (e.g., KCNK2, KCNK3, and KCNK4) have been reported to be associated with inflammatory mechanisms (8, 9, 35, 38, 45). Therefore, the increased KCNK1/KCNK2 channels may be also involved in inflammatory processes through JNK signaling pathway in PAH. Further experiments are necessary for elucidating the underlying mechanisms of JNK phosphorylation following the activation of KCNK1 and KCNK2 channels.

Due to the recent development of specific PAH drugs, the five-year survival rate of PAH after its diagnosis has increased to 60%–70% in the USA (54), UK, Ireland (55), Spain (56), and France (57). For the treatment of PAH, endothelin receptor antagonists, prostacyclin analogues, a prostaglandin I_2_ receptor agonist, phosphodiesterase type 5 inhibitors, and a soluble guanylate cyclase stimulator have been approved (1). Nevertheless, PAH remains incurable and still has a poor prognosis. In the medical management of PAH, monotherapy with an approved drug is used to treat low-risk PAH patients. Since the clinical response is occasionally insufficient, combination therapy using two or three approved drugs with different mechanisms of action is initiated. Combination therapy is also used for intermediate- or high-risk PAH patients (1). Therefore, novel targets for specific PAH drugs are required in therapeutic strategies for PAH (58).

KCNK2 channels are expected to become an interactive target for the treatment of depression, cerebral ischemia, general anesthesia, analgesics, ventricular tachycardia, and cancer (35). A recent study reported that treprostinil (prostacyclin analogue), which is used for PAH patients, inhibited KCNK2 channels (59). The effects of treprostinil in PAH patients may be partially mediated by its inhibition of KCNK2 channels. On the other hand, KCNK1 channels may be a molecular target for the treatment of cardiac arrhythmia and cancer (9). The present investigation demonstrated the up-regulated expression of KCNK1/TWIK1 and KCNK2/TREK1 channels in PASMCs from IPAH patients and experimental PH animals, which may be involved in vascular remodeling in PAH. This information provides insights into the underlying mechanisms of PAH and will lead to the development of novel PAH drugs.

## Data Availability

The raw data supporting the conclusions of this article will be made available by the authors, without undue reservation.

## References

[1] Hassoun PM. Pulmonary arterial hypertension. N Engl J Med. (2021) 385:2361–76. 10.1056/NEJMra2000348 34910865

[2] Badesch DB Raskob GE Elliott CG Krichman AM Farber HW Frost AE Pulmonary arterial hypertension: baseline characteristics from the REVEAL registry. Chest. (2010) 137:376–87. 10.1378/chest.09-1140 19837821

[3] Southgate L Machado RD Gräf S Morrell NW. Molecular genetic framework underlying pulmonary arterial hypertension. Nat Rev Cardiol. (2020) 17:85–95. 10.1038/s41569-019-0242-x 31406341

[4] Morrell NW Adnot S Archer SL Dupuis J Lloyd Jones P Maclean MR Cellular and molecular basis of pulmonary arterial hypertension. J Am Coll Cardiol. (2009) 54:S20–31. 10.1016/j.jacc.2009.04.018 19555855 PMC2790324

[5] Kuhr FK Smith KA Song MY Levitan I Yuan JX. New mechanisms of pulmonary arterial hypertension: role of Ca^2+^ signaling. Am J Physiol Heart Circ Physiol. (2012) 302:H1546–62. 10.1152/ajpheart.00944.2011 22245772 PMC3330808

[6] Olschewski A Papp R Nagaraj C Olschewski H. Ion channels and transporters as therapeutic targets in the pulmonary circulation. Pharmacol Ther. (2014) 144:349–68. 10.1016/j.pharmthera.2014.08.001 25108211

[7] Lambert M Capuano V Olschewski A Sabourin J Nagaraj C Girerd B Ion channels in pulmonary hypertension: a therapeutic interest? Int J Mol Sci. (2018) 19:3162. 10.3390/ijms19103162 30322215 PMC6214085

[8] Enyedi P Czirják G. Molecular background of leak K^+^ currents: two-pore domain potassium channels. Physiol Rev. (2010) 90:559–605. 10.1152/physrev.00029.2009 20393194

[9] Feliciangeli S Chatelain FC Bichet D Lesage F. The family of K_2P_ channels: salient structural and functional properties. J Physiol. (2015) 593:2587–603. 10.1113/jphysiol.2014.287268 25530075 PMC4500345

[10] Wiedmann F Frey N Schmidt C. Two-pore-domain potassium (K_2P_-) channels: cardiac expression patterns and disease-specific remodelling processes. Cells. (2021) 10:2914. 10.3390/cells10112914 34831137 PMC8616229

[11] Awad KS West JD De Jesus Perez V Maclean M. Novel signaling pathways in pulmonary arterial hypertension (2015 grover conference series). Pulm Circ. (2016) 6:285–94. 10.1086/688034 27683605 PMC5019081

[12] Jin N Hatton N Swartz DR Xia X Harrington MA Larsen SH Hypoxia activates jun-N-terminal kinase, extracellular signal-regulated protein kinase, and p38 kinase in pulmonary arteries. Am J Respir Cell Mol Biol. (2000) 23:593–601. 10.1165/ajrcmb.23.5.3921 11062137

[13] Henriques-Coelho T Oliveira SM Moura RS Roncon-Albuquerque R Jr. Neves AL Santos M Thymulin inhibits monocrotaline-induced pulmonary hypertension modulating interleukin-6 expression and suppressing p38 pathway. Endocrinology. (2008) 149:4367–73. 10.1210/en.2008-0018 18511508

[14] Wilson JL Yu J Taylor L Polgar P. Hyperplastic growth of pulmonary artery smooth muscle cells from subjects with pulmonary arterial hypertension is activated through JNK and p38 MAPK. PLoS One. (2015) 10:e0123662. 10.1371/journal.pone.0123662 25905460 PMC4408087

[15] Guo M Zhang M Cao X Fang X Li K Qin L Notch4 mediates vascular remodeling via ERK/JNK/P38 MAPK signaling pathways in hypoxic pulmonary hypertension. Respir Res. (2022) 23:6. 10.1186/s12931-022-01927-9 35016680 PMC8753901

[16] Das M Zawada WM West J Stenmark KR. JNK2 Regulates vascular remodeling in pulmonary hypertension. Pulm Circ. (2018) 8:1–13. 10.1177/2045894018778156 PMC6055330 29718758

[17] Sala MA Chen C Zhang Q Do-Umehara HC Wu W Misharin AV JNK2 up-regulates hypoxia-inducible factors and contributes to hypoxia-induced erythropoiesis and pulmonary hypertension. J Biol Chem. (2018) 293:271–84. 10.1074/jbc.RA117.000440 29118187 PMC5766905

[18] Yuan JX Aldinger AM Juhaszova M Wang J Conte JV Jr. Gaine SP Dysfunctional voltage-gated K^+^ channels in pulmonary artery smooth muscle cells of patients with primary pulmonary hypertension. Circulation. (1998) 98:1400–6. 10.1161/01.CIR.98.14.1400 9760294

[19] Yu Y Fantozzi I Remillard CV Landsberg JW Kunichika N Platoshyn O Enhanced expression of transient receptor potential channels in idiopathic pulmonary arterial hypertension. Proc Natl Acad Sci U S A. (2004) 101:13861–6. 10.1073/pnas.0405908101 15358862 PMC518765

[20] Stenmark KR Meyrick B Galie N Mooi WJ Mcmurtry IF. Animal models of pulmonary arterial hypertension: the hope for etiological discovery and pharmacological cure. Am J Physiol Lung Cell Mol Physiol. (2009) 297:L1013–32. 10.1152/ajplung.00217.2009 19748998

[21] Maarman G Lecour S Butrous G Thienemann F Sliwa K. A comprehensive review: the evolution of animal models in pulmonary hypertension research; are we there yet? Pulm Circ. (2013) 3:739–56. 10.1086/674770 25006392 PMC4070827

[22] Dignam JP Scott TE Kemp-Harper BK Hobbs AJ. Animal models of pulmonary hypertension: getting to the heart of the problem. Br J Pharmacol. (2022) 179:811–37. 10.1111/bph.15444 33724447

[23] Kawade A Yamamura A Kondo R Suzuki Y Yamamura H. Corosolic acid ameliorates vascular remodeling in pulmonary arterial hypertension via the downregulation of STAT3 signaling. J Pharmacol Sci. (2023) 151:119–27. 10.1016/j.jphs.2022.12.007 36707177

[24] Kawade A Yamamura A Fujiwara M Kobayashi S Mori S Horii C Comparative analysis of age in monocrotaline-induced pulmonary hypertensive rats. J Pharmacol Sci. (2021) 147:81–5. 10.1016/j.jphs.2021.05.012 34294376

[25] Ishida M Yamamura A Fujiwara M Amano T Ota M Hikawa Y Pimaric acid reduces vasoconstriction via BK_Ca_ channel activation and VDCC inhibition in rat pulmonary arterial smooth muscles. J Pharmacol Sci. (2023) 153:84–8. 10.1016/j.jphs.2023.08.001 37640473

[26] Yamamura A Guo Q Yamamura H Zimnicka AM Pohl NM Smith KA Enhanced Ca^2+^-sensing receptor function in idiopathic pulmonary arterial hypertension. Circ Res. (2012) 111:469–81. 10.1161/CIRCRESAHA.112.266361 22730443 PMC3695473

[27] Amano T Yamamura A Fujiwara M Hirai S Kondo R Suzuki Y Upregulated ClC3 channels/transporters elicit swelling-activated Cl^−^ currents and induce excessive cell proliferation in idiopathic pulmonary arterial hypertension. Biol Pharm Bull. (2022) 45:1684–91. 10.1248/bpb.b22-00513 35989293

[28] Zhou M Xu G Xie M Zhang X Schools GP Ma L TWIK-1 and TREK-1 are potassium channels contributing significantly to astrocyte passive conductance in rat hippocampal slices. J Neurosci. (2009) 29:8551–64. 10.1523/JNEUROSCI.5784-08.2009 19571146 PMC6665656

[29] Decher N Rinné S Bedoya M Gonzalez W Kiper AK. Molecular pharmacology of K_2P_ potassium channels. Cell Physiol Biochem. (2021) 55:87–107. 10.33594/000000339 33667333

[30] Piechotta PL Rapedius M Stansfeld PJ Bollepalli MK Ehrlich G Andres-Enguix I The pore structure and gating mechanism of K2P channels. EMBO J. (2011) 30:3607–19. 10.1038/emboj.2011.268 21822218 PMC3181484

[31] Schewe M Nematian-Ardestani E Sun H Musinszki M Cordeiro S Bucci G A non-canonical voltage-sensing mechanism controls gating in K2P K^+^ channels. Cell. (2016) 164:937–49. 10.1016/j.cell.2016.02.002 26919430 PMC4771873

[32] Kondo R Deguchi A Kawata N Suzuki Y Yamamura H. Involvement of TREK1 channels in the proliferation of human hepatic stellate LX-2 cells. J Pharmacol Sci. (2022) 148:286–94. 10.1016/j.jphs.2022.01.003 35177207

[33] Fernandez RA Wan J Song S Smith KA Gu Y Tauseef M Upregulated expression of STIM2, TRPC6, and Orai2 contributes to the transition of pulmonary arterial smooth muscle cells from a contractile to proliferative phenotype. Am J Physiol Cell Physiol. (2015) 308:C581–93. 10.1152/ajpcell.00202.2014 25673771 PMC4398853

[34] Imaizumi Y. Reciprocal relationship between Ca^2+^ signaling and Ca^2+^-gated ion channels as a potential target for drug discovery. Biol Pharm Bull. (2022) 45:1–18. 10.1248/bpb.b21-00896 34980771

[35] Djillani A Mazella J Heurteaux C Borsotto M. Role of TREK-1 in health and disease, focus on the central nervous system. Front Pharmacol. (2019) 10:379. 10.3389/fphar.2019.00379 31031627 PMC6470294

[36] Gardener MJ Johnson IT Burnham MP Edwards G Heagerty AM Weston AH. Functional evidence of a role for two-pore domain potassium channels in rat mesenteric and pulmonary arteries. Br J Pharmacol. (2004) 142:192–202. 10.1038/sj.bjp.0705691 15066906 PMC1574915

[37] Ma L Roman-Campos D Austin ED Eyries M Sampson KS Soubrier F A novel channelopathy in pulmonary arterial hypertension. N Engl J Med. (2013) 369:351–61. 10.1056/NEJMoa1211097 23883380 PMC3792227

[38] Olschewski A Veale EL Nagy BM Nagaraj C Kwapiszewska G Antigny F TASK-1 (KCNK3) channels in the lung: from cell biology to clinical implications. Eur Respir J. (2017) 50:1700754. 10.1183/13993003.00754-2017 29122916

[39] Nagaraj C Tang B Bálint Z Wygrecka M Hrzenjak A Kwapiszewska G Src tyrosine kinase is crucial for potassium channel function in human pulmonary arteries. Eur Respir J. (2013) 41:85–95. 10.1183/09031936.00211811 22523355

[40] Antigny F Hautefort A Meloche J Belacel-Ouari M Manoury B Rucker-Martin C Potassium channel subfamily K member 3 (KCNK3) contributes to the development of pulmonary arterial hypertension. Circulation. (2016) 133:1371–85. 10.1161/CIRCULATIONAHA.115.020951 26912814

[41] Lambert M Capuano V Boet A Tesson L Bertero T Nakhleh MK Characterization of Kcnk3-mutated rat, a novel model of pulmonary hypertension. Circ Res. (2019) 125:678–95. 10.1161/CIRCRESAHA.119.314793 31347976

[42] Pandit LM Lloyd EE Reynolds JO Lawrence WS Reynolds C Wehrens XH TWIK-2 channel deficiency leads to pulmonary hypertension through a rho-kinase-mediated process. Hypertension. (2014) 64:1260–5. 10.1161/HYPERTENSIONAHA.114.03406 25245387 PMC4231005

[43] Temple IP Logantha SJ Absi M Zhang Y Pervolaraki E Yanni J Atrioventricular node dysfunction and ion channel transcriptome in pulmonary hypertension. Circ Arrhythm Electrophysiol. (2016) 9:e003432. 10.1161/CIRCEP.115.003432 27979911 PMC5679363

[44] Lambert M Boet A Rucker-Martin C Mendes-Ferreira P Capuano V Hatem S Loss of KCNK3 is a hallmark of RV hypertrophy/dysfunction associated with pulmonary hypertension. Cardiovasc Res. (2018) 114:880–93. 10.1093/cvr/cvy016 29360952

[45] Saint-Martin Willer A Santos-Gomes J Adão R Brás-Silva C Eyries M Pérez-Vizcaino F Physiological and pathophysiological roles of the KCNK3 potassium channel in the pulmonary circulation and the heart. J Physiol. (2023) 601:3717–37. 10.1113/JP284936 37477289

[46] Smith KA Voiriot G Tang H Fraidenburg DR Song S Yamamura H Notch activation of Ca^2+^ signaling in the development of hypoxic pulmonary vasoconstriction and pulmonary hypertension. Am J Respir Cell Mol Biol. (2015) 53:355–67. 10.1165/rcmb.2014-0235OC 25569851 PMC4566064

[47] Tang H Yamamura A Yamamura H Song S Fraidenburg DR Chen J Pathogenic role of calcium-sensing receptors in the development and progression of pulmonary hypertension. Am J Physiol Lung Cell Mol Physiol. (2016) 310:L846–59. 10.1152/ajplung.00050.2016 26968768 PMC4867349

[48] Song S Yamamura A Yamamura H Ayon RJ Smith KA Tang H Flow shear stress enhances intracellular Ca^2+^ signaling in pulmonary artery smooth muscle cells from patients with pulmonary arterial hypertension. Am J Physiol Cell Physiol. (2014) 307:C373–83. 10.1152/ajpcell.00115.2014 24920677 PMC4137136

[49] Song S Ayon RJ Yamamura A Yamamura H Dash S Babicheva A Capsaicin-induced Ca^2+^ signaling is enhanced via upregulated TRPV1 channels in pulmonary artery smooth muscle cells from patients with idiopathic PAH. Am J Physiol Lung Cell Mol Physiol. (2017) 312:L309–25. 10.1152/ajplung.00357.2016 27979859 PMC5374303

[50] Yamamura A Yamamura H Guo Q Zimnicka AM Wan J Ko EA Dihydropyridine Ca^2+^ channel blockers increase cytosolic [Ca^2+^] by activating Ca^2+^-sensing receptors in pulmonary arterial smooth muscle cells. Circ Res. (2013) 112:640–50. 10.1161/CIRCRESAHA.113.300897 23300272 PMC3642037

[51] Miyaki R Yamamura A Kawade A Fujiwara M Kondo R Suzuki Y SKF96365 activates calcium-sensing receptors in pulmonary arterial smooth muscle cells. Biochem Biophys Res Commun. (2022) 607:44–8. 10.1016/j.bbrc.2022.03.121 35366542

[52] Babicheva A Ayon RJ Zhao T Ek Vitorin JF Pohl NM Yamamura A MicroRNA-mediated downregulation of K^+^ channels in pulmonary arterial hypertension. Am J Physiol Lung Cell Mol Physiol. (2020) 318:L10–26. 10.1152/ajplung.00010.2019 31553627 PMC6985878

[53] Abraham DM Lee TE Watson LJ Mao L Chandok G Wang HG The two-pore domain potassium channel TREK-1 mediates cardiac fibrosis and diastolic dysfunction. J Clin Invest. (2018) 128:4843–55. 10.1172/JCI95945 30153110 PMC6205385

[54] Benza RL Miller DP Barst RJ Badesch DB Frost AE Mcgoon MD. An evaluation of long-term survival from time of diagnosis in pulmonary arterial hypertension from the REVEAL registry. Chest. (2012) 142:448–56. 10.1378/chest.11-1460 22281797

[55] Ling Y Johnson MK Kiely DG Condliffe R Elliot CA Gibbs JS Changing demographics, epidemiology, and survival of incident pulmonary arterial hypertension: results from the pulmonary hypertension registry of the United Kingdom and Ireland. Am J Respir Crit Care Med. (2012) 186:790–6. 10.1164/rccm.201203-0383OC 22798320

[56] Escribano-Subias P Blanco I López-Meseguer M Lopez-Guarch CJ Roman A Morales P Survival in pulmonary hypertension in Spain: insights from the spanish registry. Eur Respir J. (2012) 40:596–603. 10.1183/09031936.00101211 22362843

[57] Boucly A Savale L Jaïs X Bauer F Bergot E Bertoletti L Association between initial treatment strategy and long-term survival in pulmonary arterial hypertension. Am J Respir Crit Care Med. (2021) 204:842–54. 10.1164/rccm.202009-3698OC 34185620

[58] Sommer N Ghofrani HA Pak O Bonnet S Provencher S Sitbon O Current and future treatments of pulmonary arterial hypertension. Br J Pharmacol. (2021) 178:6–30. 10.1111/bph.15016 32034759

[59] Cunningham KP Clapp LH Mathie A Veale EL. The prostacyclin analogue, treprostinil, used in the treatment of pulmonary arterial hypertension, is a potent antagonist of TREK-1 and TREK-2 potassium channels. Front Pharmacol. (2021) 12:705421. 10.3389/fphar.2021.705421 34267666 PMC8276018

