# Supplementary material for: Up-regulated expression of two-pore domain K+ channels, KCNK1 and KCNK2, is involved in the proliferation and migration of pulmonary arterial smooth muscle cells in pulmonary arterial hypertension
Source: Front Cardiovasc Med. 2024 Feb 12;11:1343804. doi: 10.3389/fcvm.2024.1343804 (PMC10894933; doi:10.3389/fcvm.2024.1343804)
Supplement: Supplementary file 1 [file Table1.docx]

**Supplemental Table S1.**

**Expression ratios of KCNK channels in IPAH-PASMCs to normal-PASMCs.**

| Gene | mRNA expression (ratio to β-actin) | | P value  (Normal vs. IPAH) | Ratio  (IPAH/Normal) |
| --- | --- | --- | --- | --- |
|  | Normal | IPAH |  |  |
| KCNK1 (TWIK1) | 0.000018±0.000005 | 0.000612±0.000124 | 0.029* | 34.699±7.048 |
| KCNK2 (TREK1) | 0.001543±0.000140 | 0.003188±0.000537 | 0.029* | 2.066±0.348 |
| KCNK3 (TASK1) | 0.000246±0.000029 | 0.000064±0.000035 | 0.029* | 0.262±0.144 |
| KCNK4 (TRAAK) | 0.000027±0.000006 | 0.000028±0.000013 | 0.686 | 1.042±0.486 |
| KCNK5 (TASK2) | 0.000006±0.000002 | 0.000011±0.000007 | 0.886 | 1.884±1.119 |
| KCNK6 (TWIK2) | 0.011853±0.000827 | 0.003658±0.000339 | 0.029* | 0.309±0.029 |
| KCNK7 | 0.000118±0.000011 | 0.000100±0.000014 | 0.200 | 0.847±0.117 |
| KCNK9 (TASK3) | 0.000004±0.000001 | 0.000120±0.000095 | 0.057 | 27.834±21.855 |
| KCNK10 (TREK2) | 0.000004±0.000003 | 0.000003±0.000002 | 1.000 | 0.626±0.367 |
| KCNK12 (THIK2) | 0.000005±0.000001 | 0.000033±0.000005 | 0.029* | 6.798±0.950 |
| KCNK13 (THIK1) | 0.000008±0.000001 | 0.000022±0.000006 | 0.029* | 2.965±0.748 |
| KCNK15 (TASK5) | 0.000025±0.000013 | 0.000007±0.000002 | 0.343 | 0.294±0.079 |
| KCNK16 (TALK1) | 0.000037±0.000020 | 0.000012±0.000003 | 0.343 | 0.332±0.079 |
| KCNK17 (TALK2) | 0.000025±0.000006 | 0.000015±0.000003 | 0.343 | 0.593±0.125 |
| KCNK18 (TRESK) | 0.000008±0.000005 | 0.000002±0.000001 | 0.057 | 0.245±0.085 |

These data were used to make the bar graph in Figure 1A. Data are presented as means±S.E. (n=4). The significance of differences between two groups was examined using the non-parametric Mann-Whitney U test (*p<0.05).
