# Supplementary material for: Up-regulated expression of two-pore domain K+ channels, KCNK1 and KCNK2, is involved in the proliferation and migration of pulmonary arterial smooth muscle cells in pulmonary arterial hypertension
Source: Front Cardiovasc Med. 2024 Feb 12;11:1343804. doi: 10.3389/fcvm.2024.1343804 (PMC10894933; doi:10.3389/fcvm.2024.1343804)
Supplement: Supplementary file 2 [file Table2.docx]

**Supplemental Table S2.**

**Expression of KCNK channels in right ventricle from MCT-PH rats.**

| Gene | mRNA expression (ratio to β-actin) | | P value |
| --- | --- | --- | --- |
|  | Control | MCT |  |
| Kcnk1 (TWIK1) | 0.000535±0.000076 | 0.000589±0.000099 | 1.000 |
| Kcnk2 (TREK1) | 0.001227±0.000131 | 0.004660±0.001050 | 0.038* |
| Kcnk3 (TASK1) | 0.016200±0.003763 | 0.009003±0.000507 | 0.017* |
| Kcnk6 (TWIK2) | 0.003227±0.001008 | 0.002477±0.000343 | 0.875 |

Data are presented as means±S.E. (n=7). The significance of differences between

two groups was examined using the non-parametric Mann-Whitney U test (*p<0.05).
